# Alterations in Intestinal Microbiota of Children With Celiac Disease at the Time of Diagnosis and on a Gluten-free Diet

**DOI:** 10.1053/j.gastro.2020.08.007

**Published:** 2020-12

**Authors:** Konstantina Zafeiropoulou, Ben Nichols, Mary Mackinder, Olga Biskou, Eleni Rizou, Antonia Karanikolou, Clare Clark, Elaine Buchanan, Tracey Cardigan, Hazel Duncan, David Wands, Julie Russell, Richard Hansen, Richard K. Russell, Paraic McGrogan, Christine A. Edwards, Umer Z. Ijaz, Konstantinos Gerasimidis

**Affiliations:** 1 Human Nutrition, School of Medicine, Dentistry and Nursing, College of Medical, Veterinary and Life Sciences, University of Glasgow, Glasgow Royal Infirmary, Glasgow, Scotland, UK; 2 Department of Paediatric Gastroenterology, Royal Hospital for Children, Glasgow, Scotland, UK; 3 Civil Engineering, School of Engineering, University of Glasgow, Glasgow, Scotland, UK

**Keywords:** OTU, Pediatric, Microbiome, Short-chain Fatty Acids, BCFA, branch-chain fatty acid, CD, celiac disease, GFD, gluten-free diet, GI, gastrointestinal, GIP, gluten immunogenic peptide, HC, healthy control subjects, OTU, operational taxonomic unit, SCFA, short-chain fatty acid, TCD, treated celiac disease, UCD, untreated celiac disease

## Abstract

**Background And Aims:**

It is not clear whether alterations in the intestinal microbiota of children with celiac disease (CD) cause the disease or are a result of disease and/or its treatment with a gluten-free diet (GFD).

**Methods:**

We obtained 167 fecal samples from 141 children (20 with new-onset CD, 45 treated with a GFD, 57 healthy children, and 19 unaffected siblings of children with CD) in Glasgow, Scotland. Samples were analyzed by 16S ribosomal RNA sequencing, and diet-related metabolites were measured by gas chromatography. We obtained fecal samples from 13 children with new-onset CD after 6 and 12 months on a GFD. Relationships between microbiota with diet composition, gastrointestinal function, and biomarkers of GFD compliance were explored.

**Results:**

Microbiota α diversity did not differ among groups. Microbial dysbiosis was not observed in children with new-onset CD. In contrast, 2.8% (Bray-Curtis dissimilarity index, *P* = .025) and 2.5% (UniFrac distances, *P* = .027) of the variation in microbiota composition could be explained by the GFD. Between 3% and 5% of all taxa differed among all group comparisons. Eleven distinctive operational taxonomic units composed a microbe signature specific to CD with high diagnostic probability. Most operational taxonomic units that differed between patients on a GFD with new-onset CD vs healthy children were associated with nutrient and food group intake (from 75% to 94%) and with biomarkers of gluten ingestion. Fecal levels of butyrate and ammonia decreased during the GFD.

**Conclusions:**

Although several alterations in the intestinal microbiota of children with established CD appear to be effects of a GFD, specific bacteria were found to be distinct biomarkers of CD. Studies are needed to determine whether these bacteria contribute to pathogenesis of CD.

## Graphical abstract

**Figure:**
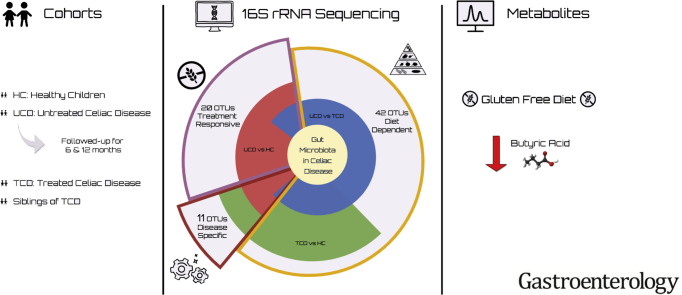

See Covering the Cover synopsis on page 1995.

**What You Need to Know**

**Background and Context**

It is not clear whether alterations in the intestinal microbiota of children with celiac disease cause the disease or are a result of disease and/or its treatment with gluten-free diet (GFD).

**New Findings**

Although several alterations in the intestinal microbiota of children with established celiac disease appear to be effects of a GFD, there are specific bacteria that are distinct biomarkers of celiac disease.

**Limitations**

It is not clear whether the microbes identified contribute to pathogenesis of celiac disease or are the result of it.

**Impact**

The GFD alters the intestinal microbiota, but in patients with celiac disease, there are additional differences, compared with healthy children.

Celiac disease (CD) is an autoimmune destruction of small intestinal villi triggered by ingestion of gluten in genetically susceptible individuals.^1^ It causes nutrient malabsorption, leading to intestinal and extraintestinal symptoms.^2^ Lifelong adherence to a gluten-free diet (GFD) is the only treatment. The underlying pathogenesis of CD is multifactorial, and although genetic predisposition occurs in 30%–40% of the general population, only a small proportion of these individuals will develop CD, suggesting environmental factors are at play.^3^ This premise is supported by the rise in the incidence of CD over past decades, suggesting that changes in population genetics and changes in gluten consumption cannot explain this increase.^3^ Weaning practices, antibiotic exposure,^4^^,^^5^ and viral gastrointestinal (GI) infections^6^ have been implicated as risk factors for CD onset,^7^ Evidence points to the involvement of the gut microbiota,^8^, ^9^, ^10^, ^11^, ^12^, ^13^, ^14^, ^15^, ^16^, ^17^ driven by the rapid expansion of microbiota research.

Several noncommunicable diseases, like inflammatory bowel disease, show increasing incidence similar to CD and have been associated with distinct features of microbiota structure and function, also termed “dysbiosis.”^18^ There has been increasing interest in research exploring the role of the microbiota in CD. However, results remain inconclusive. Previous research was mostly of a cross-sectional design providing a snapshot of the gut microbiota in CD and, most importantly, did not determine whether a disturbed microbiota in feces of new-onset, untreated patients (untreated celiac disease [UCD]) was implicated in disease pathogenesis or was predominantly an epiphenomenon of the underlying disease, altered GI motility, and excessive substrate availability because of nutrient malabsorption and intestinal inflammation. Research has also described an altered microbiota in treated patients with CD (TCD) but did not explore the extent to which these signals were attributed to dietary modification and exclusions imposed during treatment with a GFD. Studies describing the human fecal microbiota of CD using next-generation sequencing do not exist, and previous research relied on characterization of selective microbial groups.^8^, ^9^, ^10^, ^11^, ^12^, ^13^, ^14^, ^15^, ^16^, ^17^

This study characterized the gut microbiota of children with CD. We combined cross-sectional and prospective patient cohorts and collected data on determinants of the microbiota likely to change in children with CD treated with a GFD or likely to differ when comparing CD with healthy control subjects (HCs). For the first time in the literature, we profiled the fecal microbiota using 16S ribosomal RNA gene sequencing and measured metabolites likely to be influenced by adherence to a GFD. Additionally, we explored associations with dietary intake, GI symptoms, and novel biomarkers of GFD compliance. We hypothesized that the gut microbiota of CD patients differs from HCs; several of these microbial signals are secondary effects of dietary modifications during a GFD, but others may be implicated in the underlying disease pathogenesis.

## Material and Methods

### Subjects

Fecal samples were collected from children with CD attending the Royal Hospital for Children in Glasgow. New-onset patients with CD were referred from primary healthcare services, whereas previously diagnosed children were recruited from clinics where patients attend annually. Siblings of CD children with no clinical symptoms and negative tissue transglutaminase IgA antibodies and healthy volunteers recruited via advertisement were used for comparative analysis. In these 2 groups, healthy status was defined as children who did not visit their general practitioner for a medical condition regularly or who did not receive regular medication and who had no past history of chronic GI disorders. All consecutive children were invited to participate unless they met 1 of the exclusion criteria (ie, antibiotic use or regular use of probiotics/prebiotics in the preceding 3 months). Patients with other comorbidities were also excluded.

CD was confirmed by small bowel biopsy using UK guidelines in place at the time of recruitment.^19^ Dietary intake (macronutrient and food groups) was evaluated using a Scottish food frequency questionnaire.^20^ GI symptoms were evaluated using the PedsQL-GS questionnaire (version 1).^21^ The higher the PedsQL-GS score, the lower the GI symptoms. Recent gluten ingestion, a proxy marker of GFD compliance, was evaluated by measuring the fecal gluten immunogenic peptide (GIP) levels (iVYLISA; Biomedal, Seville, Spain).^22^

### Fecal Sample Collection

The entire bowel movement was collected, stored under anaerobic (Anaerocult A; Merck, Darmstadt, Germany) cold conditions, and processed within 2 hours of defecation. The entire sample was homogenized with a hand blender and stored appropriately for downstream analysis. A single sample was collected from all groups apart from the UCD patients. For the UCD group we collected 3 samples: 1 baseline sample before diagnostic endoscopy while the patients were on a gluten-containing diet and again at 6 and 12 months while patients were on a GFD.

### Fecal Water Content, pH, and Ammonia

Fecal pH was measured in aqueous slurries and fecal ammonia with an analyzer (HI 93715; Hanna Instruments, Bedfordshire, UK).^23^ Fecal water content was calculated after lyophilization.

### Microbiota Profiling

Genomic DNA was isolated using the chaotropic method within 3 months of sample collection.^24^ Sequencing of the V4 region of the 16S ribosomal RNA gene was performed with MiSeq (Illumina, San Diego, CA) using the Golay barcodes on the reverse strand.^18^^,^^25^ Barcoded amplicons were purified using the Zymoclean Gel DNA Recovery Kit (D4001, Zymo Research, Irvine, CA).

### Fecal Short-chain Fatty Acids, Lactate, and Sulfide

The short-chain fatty acids (SCFAs) acetic, propionic, butyric, and valeric acids and the branch-chain fatty acids (BCFA) isobutyric and isovaleric acids were measured using gas chromatography (Agilent 7820A; Agilent Technologies, Stockport, USA) with a DB–WAX UI column on diethyl ether extracts.^23^^,^^25^ Nitrogen was the carrier gas. D- and L-lactate were measured using a commercial kit (D, L Lactic Acid, cat no. 11112821035; R-Biopharm AG, Darmstadt, Germany) scaled down for use in a 96-well plate. Free and total sulfide were measured colorimetrically.^23^

### Bioinformatics Analysis

Quality trimming was done using Sickle, applying a sliding window approach, and trimming regions where average base quality (PHRED score) dropped below 20.^26^ Assembly of paired reads was done using PANDAseq.^27^ USEARCH was used for dereplication and clustering of sequences into operational taxonomic units (OTUs) of 97% similarity as described in https://docs.google.com/document/d/1BcZAk28k7Uycr7iKKAVSiZ0MB9jDs9bODpdPZtYFH3Y/pub#h.agz7rwlf8m6. Chimera detection involved de novo and reference-based steps, using the ChimeraSlayer gold database (http://drive5.com/uchime/uchime_download.html) derived from the ChimeraSlayer reference database (http://microbiomeutil.sourceforge.net/). OTUs were taxonomically classified using QIIME with SILVA (Max Planck Institute for Marine Microbiology and Jacobs University, Bremen, Germany) reference database (version 123). An approximately maximum-likelihood phylogenetic tree was produced using the “ginsi” alignment algorithm in MAFFT^28^ followed by FastTree.^29^

### Statistical Analysis

General linear models on Box-Cox transformed data were used to compare groups, accounting for the paired design of the prospective cohort, and adjusted using Bonferroni correction. We first compared the fecal metabolite concentrations and microbiota between HCs and siblings of CD patients. In the absence of significant differences, we removed the sibling group from further analysis. Multivariate statistical analysis was performed in R (version 3.4.0) using the packages vegan, phyloseq, and DESeq2. Samples were rarefied to 5000 reads before calculating α diversity, measured as species richness and Shannon index. Microbial composition was assessed using nonmetric multidimensional scaling plots at the genus and OTU level based on Bray-Curtis dissimilarity indices and unweighted UniFrac distances. The former considers bacterial taxon abundance, whereas the latter considers phylogenetic distances between bacterial taxa through presence/absence, regardless of proportional representation. Permutation analysis of variance was applied using the vegan Adonis function on distance matrices (Bray-Curtis/unweighted UniFrac) with data stratified by subject to allow for repeated sampling from UCD participants during follow-up. Local contribution to β diversity analysis was performed to measure the contribution of each sample to the total OTU β diversity; samples with high local contribution to β diversity represent samples that are markedly different from the average β diversity of all study samples. Differences in OTU abundances between groups were found using the DESeq2 method, with participant identification included as a variable in the input formula for paired data. For correlations Kendall rank correlation was used. Benjamini-Hochberg correction was applied to cases of multiple testing. Analysis using the Bioenv function in vegan produced subsets of OTUs whose Euclidean distance matrices correlate maximally with the Bray-Curtis dissimilarity matrices derived from complete OTU tables, thus indicating major determinants of community structure.

Random forest analysis used OTUs that significantly differed in abundance between HCs vs both UCD and TCD patients inclusive. Models were generated using log-proportional abundances via the randomForest R package, with 10,000 decision trees used per model. Model performance was assessed using the rf.significance function in the rfUtilities R package^30^ and receiver operating characteristic analysis using the ROCR R package.^31^

### Ethical Considerations

The study was approved by the West of Scotland Research Ethics Committee (reference no. 11/WS/0006). All authors had access to the study data and reviewed and approved the final manuscript.

## Results

### Participants

One hundred forty-one children participated: 45 TCD children on a GFD, 20 UCD children on a gluten-containing diet, 19 siblings of 18 TCD children, and 57 HCs (Table 1). Thirty-three eligible participants (12 girls) declined participation; 10 other CD patients (6 girls) did not meet the inclusion criteria (ie, 3 were unable to comprehend English, 1 had developmental delay, 1 child was in foster care, 4 had type 1 diabetes, 1 had congenital hypothyroidism). There was no difference in age (P = .11) or gender (P = .87) between participants and those who declined. All healthy children who expressed an interest participated in the study.

**Table 1:** Participant Characteristics of the Cross-sectional Study and Prospective Study Cohorts

	Cross-sectional Study				Prospective Study		
	HCs (n = 57)	Siblings (n = 19)	UCD Group (n = 20)	TCD Group (n = 45)	Diagnosis (n = 13)	GFD 6 Months (n = 13)	GFD 12 Months (n = 13)
Age (*y*)	7.8 (0.41)	9.1 (0.76)	10.1 (0.70)^a^	9.3 (0.47)	9.5 (0.87)	10.1 (0.87)	10.6 (0.86)
Gender, M/F	27/30	8/11	10/10	20/25	6/7	6/7	6/7
Weight (*kg*)	29.1 (1.6)	33.7 (3.8)	33.7 (2.9)	32.3 (1.7)	30.5 (3.1)^b^	32.5 (3.3)^c^	34.8 (3.5)
Height z-score	0.29 (0.15)	0.43 (0.28)	–0.16 (0.22)	0.06 (0.16)	–0.19 (0.30)	–0.15 (0.28)	–0.20 (0.27)
<-2 SD, n (%)	1 (1.8)	0 (0)	2 (10.0)	0 (0)	2 (15.4)	1 (7.7)	1 (7.7)
Body mass index *(kg/m*^2^)	16.8 (0.34)	17.5 (0.62)	17.1 (0.58)	17.4 (0.38)	16.5 (0.67)^d^	16.6 (0.71)	17.1 (0.79)
BMI z-score	0.06 (0.15)	0.24 (0.23)	–0.23 (0.25)	0.18 (0.17)	–0.40 (0.34)	–0.45 (0.33)	–0.31 (0.33)
<-2 SD, n (%)	1 (1.8)	0 (0)	2 (10.0)	0 (0)	2 (15.4)	2 (15.4)	2 (15.4)
>2 SD, n (%)	4 (7.0)	2 (10.5)	0 (0)	3 (6.7)	0 (0)	0 (0)	0 (0)
tTG (*U/mL*)	—	—	64.8 (13.3) [9]	7.9 (3.0)^e^ [2]	68.5 (19.6)^b^ [7]	9.8 (3.3)^f^ [4]	7.7 (2.0) [4]
<7, n (%)	—	—	1 (9.1)	34 (79.1)	1 (16.7)	3 (33.3)	6 (66.7)
≥7, n (%)	—	—	10 (90.9)	9 (20.9)	5 (83.3)	6 (66.7)	3 (33.3)
GIP (*μg/g*)	—	—	3.5 (0.6) [1]	0.25 (0.06)^e^	2.95 (0.76)^b^ [1]	0.22 (0.06)	0.49 (0.23)
<0.156, n (%)	—	—	1 (5.3)	38 (84.4)	0 (0)	11 (84.6)	10 (76.9)
≥0.156, n (%)	—	—	18 (94.7)	7 (15.6)	12 (100)	2 (15.4)	3 (23.1)
PedsQL-GS score	91.4 (1.7) [1]	88.2 (2.9)	57.1 (4.8)^g^	77.5 (2.7)^h^	58.3 (6.2)^i^	67.1 (5.3)	73.6 (6.4)

NOTE. Values are mean (SEM) unless otherwise defined. General linear models for UCD and TCD groups and HCs in the cross-sectional study, and general linear models accounted for paired design in the prospective study. Box-Cox transformation with optimal λ in all but body mass index z-score; pairwise comparison with Bonferroni correction; the number of missing data is shown in brackets.

a *P* = .017 compared with HCs.

b *P* < .0001 compared with GFD 6 months and GFD 12 months.

c *P* < .0001 compared with GFD 12 months.

d *P* = .018 compared with GFD 12 months.

e *P* < .0001 compared with UCD group.

f *P* = .029 compared with GFD 12 months.

g *P* < .0001 compared with HCs.

h *P* < .0001 compared with UCD group and HCs.

i *P* = .009 compared with GFD 12 months GFD

All UCD children were recommended to follow a GFD. From the 20 UCD patients with baseline fecal samples, 13 (65%) provided follow-up samples at 6 and 12 months after GFD initiation (prospective cohort); 4 (20%) patients were lost at follow-up and 3 (15%) others provided paired samples at 12 months only but were subsequently removed to avoid bias in statistical analysis. There was no difference in age (P = .27), gender (P = .998), or body mass index z-scores (P = .63) or in α and β diversity of the baseline microbiota between the 13 patients with follow-up samples and the 7 others who did not provide all samples. In total, 167 fecal samples were collected across all groups.

After commencement of a GFD, tissue transglutaminase IgA antibody titer decreased in the UCD children (Table 1). The UCD group experienced more GI problems than TCD children and HCs. The mean PedsQL-GS score was also lower in TCD children than HCs, suggesting that TCD children had more GI symptoms than HCs. In the UCD group, GI symptoms improved only 12 months after diagnosis.

Thirty-eight of 45 TCD children (84%) had undetectable GIP, indicating at least recent compliance with a GFD; the remaining 7 TCD children (16%) had detectable levels indicating either transgression from GFD recommendations or accidental exposure to gluten (Table 1). Compared with baseline, GIP concentration was almost 13 times lower at 6 months and 6 times lower at 12 months on a GFD. At 6 and 12 months after recommendation to adhere to a GFD, of 13 UCD patients, 2 (15%) and 3 (23%) had detectable GIP.

### Fecal Microbiota Profiling

**Figure 1:**
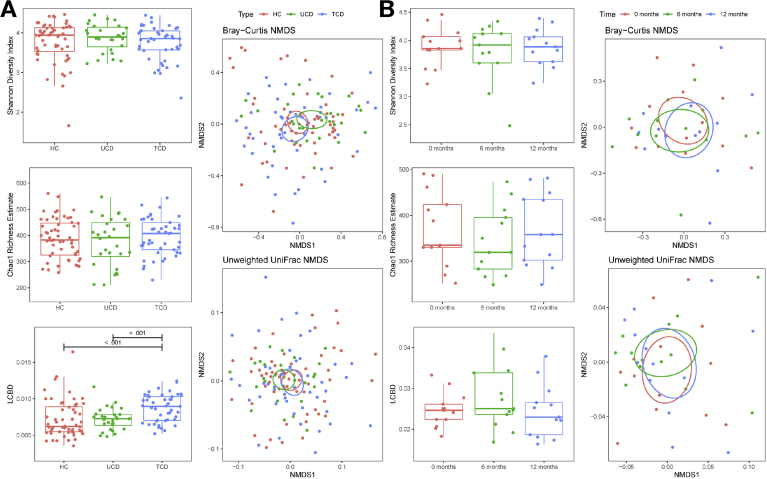
α and β Diversity for the cross-sectional (*A*) and prospective (*B*) cohort. NMDS, nonmetric multidimensional scaling.

There was no difference in α diversity between TCD, UCD, and HC groups, either at the OTU or genus level (Figure 1*A* and Supplementary Table 1). In contrast, 2.8% (P = .025) and 2.5% (P = .027) of the variation in OTU community structure (β diversity) for the Bray-Curtis dissimilarity index and unweighted UniFrac distance analyses, respectively, were explained by participant grouping. The TCD group clustered separately to HCs and tended to do so with respect to UCD (Supplementary Table 1), suggesting a significant effect of GFD on microbiota structure. Similar findings were observed at the genus level. Gender did not influence this effect. No separation in community structure was seen between the HCs and UCD group, suggesting an absence of profound dysbiosis at disease onset. Local contribution to β diversity analysis confirmed that the microbiota structure of TCD individuals differed from that of UCD children and HCs, with no difference seen between the latter 2 groups (Figure 1*A*).

Using the Bioenv workflow, a subset of 13 OTUs maintained the group clustering, explaining 92.3% of the variance described by the complete OTU dataset (Supplementary Table 2). When the Bioenv analysis was repeated for pairings of UCD vs TCD groups and HCs vs TCD group, separately, subsets of 14 and 12 OTUs were retrieved, explaining 92.0% and 91.4% of the variance described by the complete datasets, respectively. Of note, 9 OTUs featured in both pairwise comparisons, suggesting their strong influence on the fecal microbiota structure of children with CD on a GFD. Compared with disease diagnosis, there was no difference in β diversity of the 13 UCD children at 6 and 12 months after initiation of a GFD (Figure 1*B*).

### Differential Analysis in OTU Abundance Between New-onset, Untreated CD and HCs

**Figure 2:**
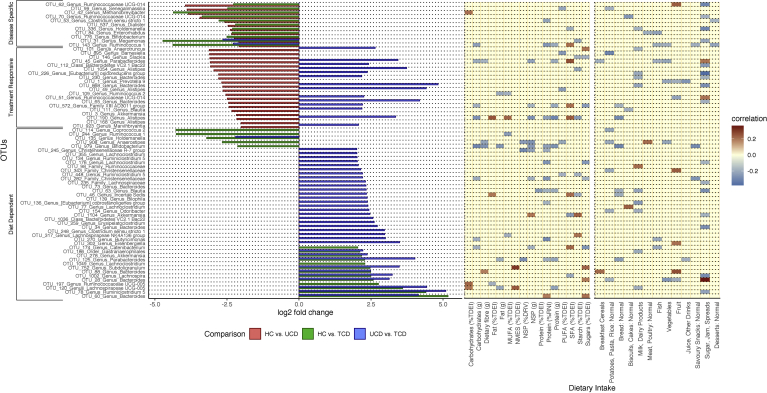
Statistically significant differences (log2-fold change) in relative abundance of OTUs between groups and correlations between these discriminatory OTUs with dietary nutrients and food groups. A negative log2-fold change represents a lower abundance in the second of the 2-group comparison.

The UCD and HC groups were characterized by 1033 distinct OTUs. Thirty-one OTUs (3%) differed significantly between the 2 groups, all of which had a significantly lower abundance in UCD children than HCs (Figure 2). Of these 31 discriminatory OTUs, only the abundance of OTU_1054 *Alistipes* correlated positively with PedsQL-GS score, suggesting the remaining 30 discriminatory OTUs were less likely to be explained by differences in GI symptoms between the 2 groups.

### Differential Analysis in OTU Abundance Between Patients on Recommendation to a GFD With Untreated CD or HCs

Next, we explored the effect a GFD might have on the CD microbiota. First, we looked for differences in OTU abundances between UCD and TCD. Fifty-one of 1082 OTUs (5%) differed significantly in abundance between the 2 groups (Figure 2 and Supplementary Table 3). Forty-eight OTUs (94%) had significantly higher abundance in TCD than UCD children apart from OTU_31 *Megamonas*, OTU_143 *Ruminococcus* 1, and OTU_135 *Holdemanella*.

Likewise, 29 of 1082 OTUs (3%) had different abundance between HCs and TCD children. Almost half (n = 13, 45%) were increased in the TCD group compared with HCs (Figure 2). Of the 13 OTUs more abundant in TCD children than HCs, 10 (77%) were significantly higher in TCD than UCD groups as well, suggesting that treatment with a GFD influences these taxa independently of disease status. Of the 16 OTUs with lower relative abundance in the TCD group than HCs, OTU_31 *Megamonas*, OTU_143 *Ruminococcus* 1, and OTU_135 *Holdemanella* had significantly lower abundance in the TCD than the UCD group (Figure 2). Of note, these 3 OTUs were the only ones with lower abundance in the TCD than the UCD group, strongly suggesting their modulation is the consequence of treatment with a GFD.

### Celiac Disease–specific Microbiota Signature

**Figure 3:**
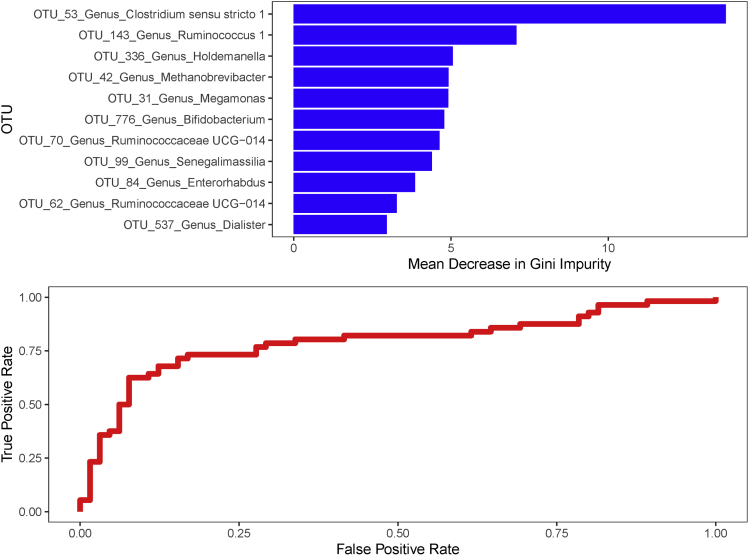
Most influential OTUs, among the 11 disease-specific, in predicting disease or health status with associated receiver operating characteristic curves and area under the curve.

**Supplementary Figure 1:**
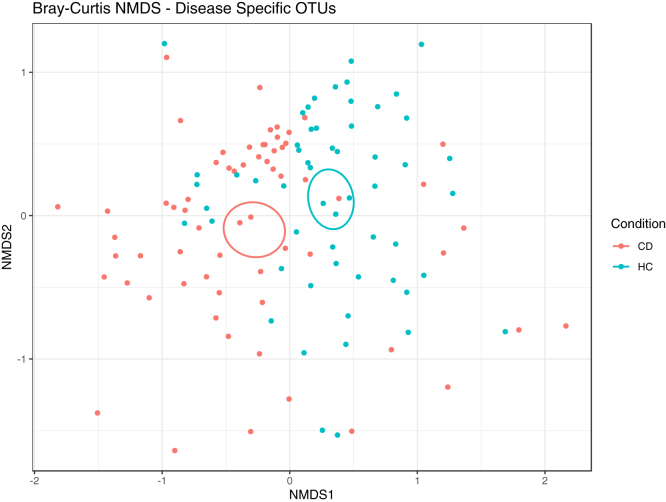
Nonmetric multidimensional scaling of the microbial community formed only by the 11 discriminant OTUs between children with CD and HCs.

Irrespective of treatment with a GFD, the relative abundance of 11 OTUs were consistently lower in children with CD than HCs (Figure 2, Supplementary Table 3), hence composing a microbial signature specific to CD. This was visualized through nonmetric multidimensional scaling analysis including only these 11 discriminant OTUs (Supplementary Figure 1). None of these 11 OTUs was associated with disease duration (median, 3.1 year; interquartile range, 1.5, 7.3) in the TCD group. Using these 11 discriminatory OTUs, random forest classifier distinguished between HCs and CD patients with an “out-of-bag” error rate of 21.5%. This was significantly more effective than random classification (permutation analysis of variance P < .001 and area under the curve, 0.789) (Figure 3). The 2 most influential OTUs were OTU_53 *Clostridium sensu stricto 1* followed by OTU_143 *Ruminococcus*.

### Discriminant Analysis in OTU Abundance in New-onset CD After Recommendation to GFD

**Figure 4:**
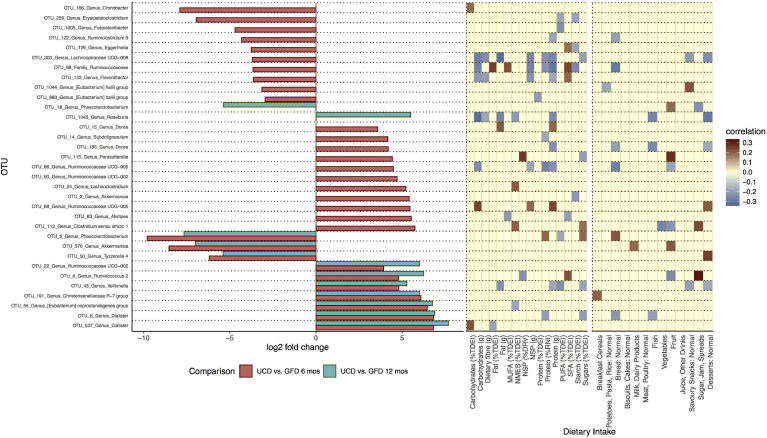
Statistically significant differences (log2-fold change) in relative abundance of OTUs between follow-up time points and correlations between these discriminatory OTUs with dietary nutrients and food groups. GFD 6 and 12 mos, UCD patients on a GFD for 6 and 12 months, respectively.

In the prospective cohort of UCD patients followed-up at 6 and 12 months on a GFD, fecal samples were characterized by 835 OTUs, 31 (3.7%) and 12 (1.4%) of which differed significantly 6 and 12 months, respectively, after initiation of a GFD (Figure 4 and Supplementary Table 4). Compared with CD diagnosis, at both 6 and 12 months on a GFD the relative abundance of 7 and 3 OTUs significantly decreased and increased, respectively. It is noteworthy that in this prospective cohort the mean effect size of a GFD on OTU abundance (Figure 4 and Supplementary Table 4) was more pronounced than the magnitude of OTU abundance difference between the TCD and UCD groups (Figure 2).

### Effect of Dietary Nutrients and Food Groups on Gut Microbiota of CD

**Supplementary Figure 2:**
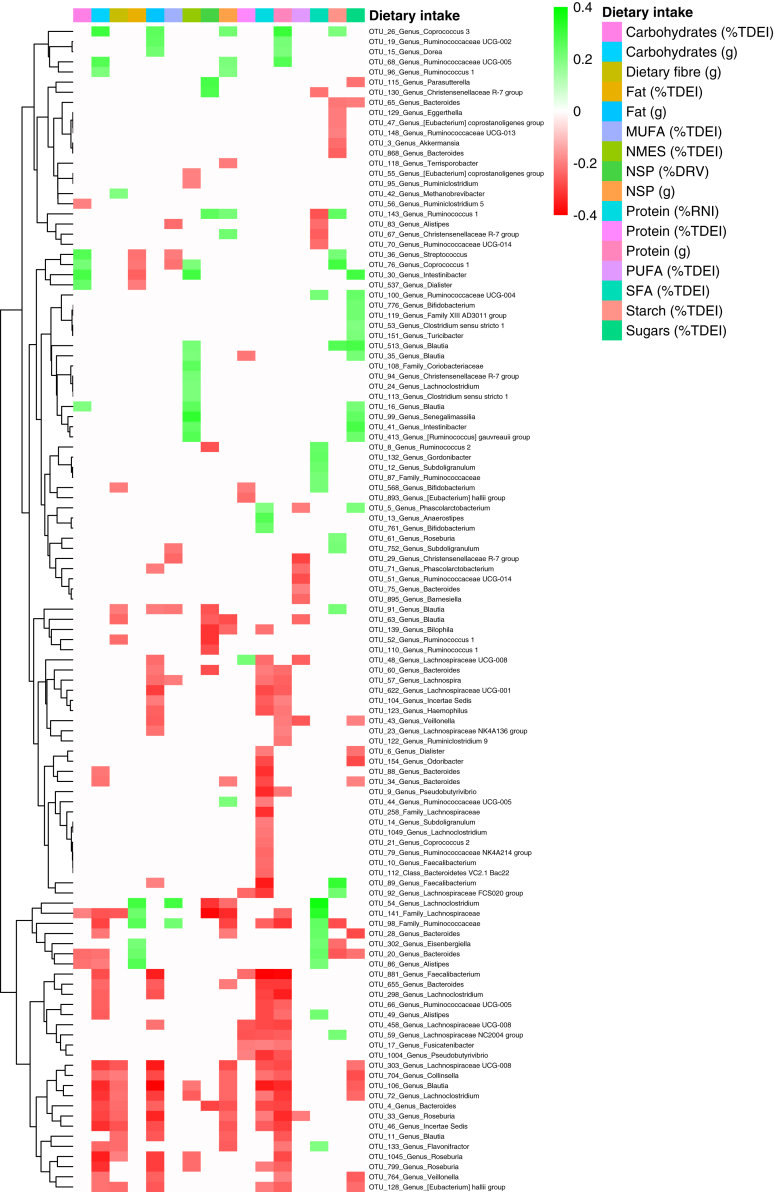
All significant correlations between the abundances of OTUs from the microbiota of HCs with their dietary nutrient intake. %TDEI, percentage of total dietary energy intake; MUFA, monounsaturated fatty acids; NMES, nonmilk extrinsic sugars; NSP, nonstarch polysaccharides; DRV, dietary reference value; RNI, reference nutrient intake; PUFA, polyunsaturated fatty acids; SFA, saturated fatty acids.

**Supplementary Figure 3:**
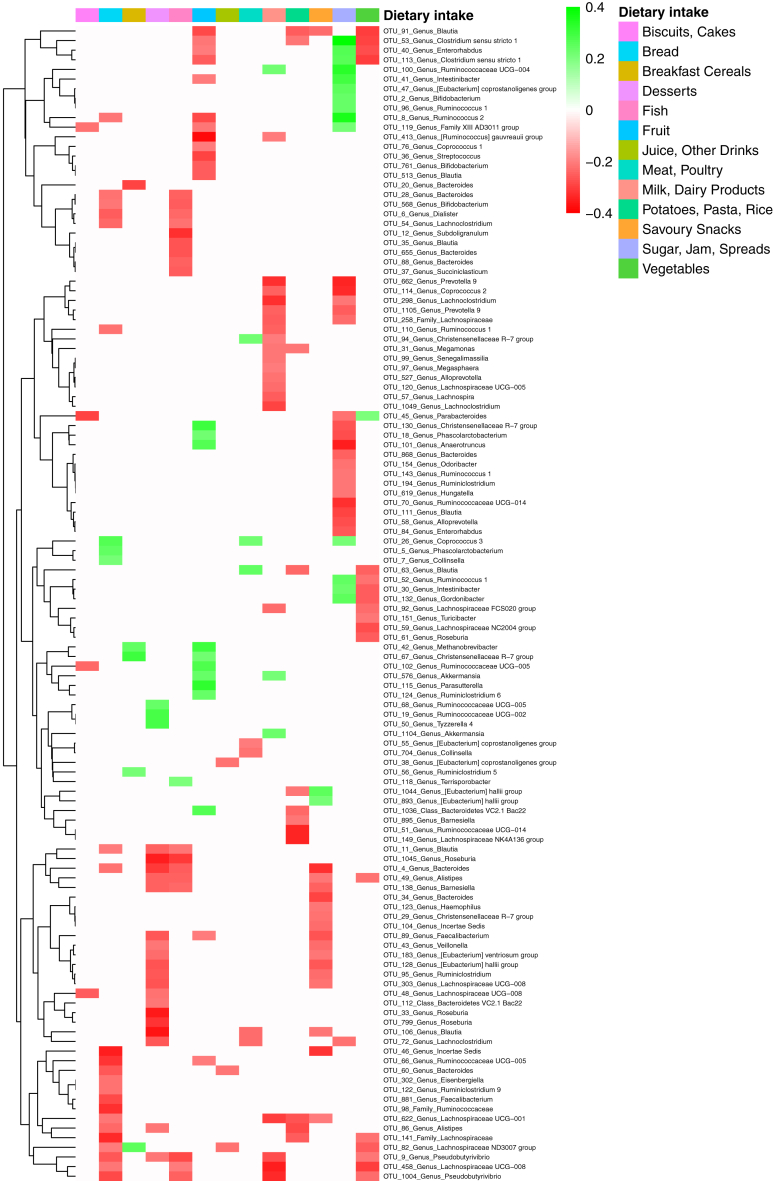
All significant correlations between the abundance of OTUs from the microbiota of HCs with the intake of food groups.

The effect dietary modifications during treatment with a GFD might have on microbiota was explored using stepwise data analysis. First, we correlated the intake of macronutrients (eg, carbohydrates) and food groups (eg, dairy portions per day) with the abundance of all OTUs characterizing the microbiota of HCs (Supplementary Figures 2 and 3). Significantly related OTUs were subsequently cross-referenced with the discriminant OTUs for comparisons between UCD children and HCs with TCD children as well as in the subset of UCD patients with paired data at 6 and 12 months. We applied this analysis workflow because, despite assessing the dietary intake of our CD patients with the food frequency questionnaire, complete nutritional composition of gluten-free products is currently unavailable and therefore the outcome of dietary assessment in this group would have been incomplete and findings misleading.

Of the 200 OTUs that associated with either the macronutrient or food group intake of HCs, 39 OTUs were differentially abundant between UCD and TCD children (Figure 2), suggesting that differences in the abundance of 39 of 51 discriminatory OTUs (76%) between UCD and TCD are likely to be explained by changes in dietary nutrient intake after a GFD recommendation. Likewise, 23 of 200 OTUs were significantly differentially abundant between HCs and TCD children (Figure 2). Therefore, differences in abundance of 23 of 29 OTUs (79%) that discriminated between HCs and TCD children are likely to be explained by changes in dietary intake after initiation of a GFD. In the prospective cohort, from the 31 and 12 OTUs whose relative abundance changed at 6 and 12 months of treatment with GFD, 29 (94%) and 11 (82%) correlated with macronutrient or food group intake in HCs (Figure 4), further supporting the hypothesis that the gut microbiota of treated CD patients differs from HCs predominantly as the result of dietary modification during a GFD.

### Differential Analysis in OTU Abundance Between Patients With and Without Recent Consumption of Gluten

**Supplementary Figure 4:**
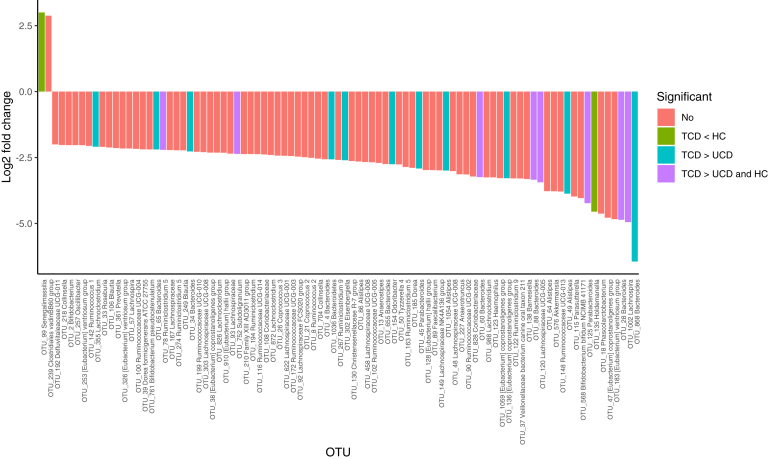
Differential OTUs between patients on a GFD recommendation with positive and negative fecal GIP. Bar color indicates significant OTUs with significant difference between 2 groups.

In pooled analysis (cross-sectional and prospective cohorts together), 12 children (17%) with recommended adherence to a GFD had detectable and 59 (83%) undetectable GIP. When we looked for differences in OTU abundance between these 2 groups, 89 OTUs differed (Supplementary Figure 4). Among these, all but 2 OTUs (OTU_99 *Senegalimassilia* and OTU_239 *Clostridiales* vadinBB60 group) were higher in children with undetectable fecal GIP.

For a few discriminatory OTUs between UCD children and HCs with TCD the direction of their change in abundance differed from that of those OTUs that discriminated between patients with and without recent gluten consumption. Nineteen of 87 OTUs (22%) with a higher abundance in children without recent gluten ingestion were significantly increased in TCD compared with UCD, with 8 of them (9%) also significantly increased in TCD children over HCs (Supplementary Figure 4). Similarly, 1 of 2 OTUs (50%) with lower abundance in children without recent gluten ingestion was also significantly lower in TCD children than in HCs.

### Comparison in Microbiota Between Treated Patients With CD and Their Unaffected Siblings

There was no difference in the microbiota structure (β diversity) of TCD patients and their unaffected siblings (Supplementary Table 1). The microbiota structure of the unaffected siblings also did not differ from HCs. Fifty-six of 964 OTUs (6%) were differentially abundant between TCD children and their unaffected siblings, with 36 (64%) significantly decreased in the TCD group (Supplementary Table 5).

### Diet-related Microbiota Metabolites

**Figure 5:**
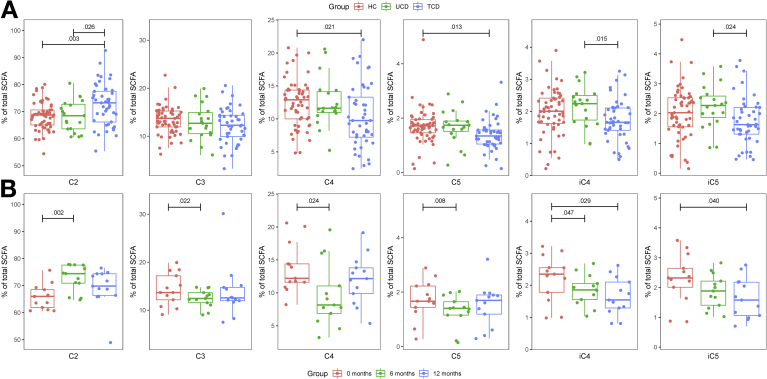
Relative proportion (%) of SCFAs. C2, acetic acid; C3, propionic acid; C4, butyric acid; C5, valeric acid; iC4, isobutyric acid; iC5, isovaleric acid.

There was no difference between the unaffected siblings of TCD children and HCs in all bacterial metabolites assayed, water content, and pH in feces (Table 2). Also, no difference was found in fecal water content and the absolute concentration of SCFAs and BCFAs among the UCD and TCD groups and HCs. However, the relative abundance (%) of acetic acid was higher and that of butyric and valeric acids lower in TCD patients than HCs (Figure 5*A*).

**Table 2:** Fecal Characteristics and Microbiota Metabolites in the Cross-sectional Study and Prospective Study Cohorts

	Cross-sectional Study				Prospective Study		
	HCs (n = 57)	Sibling (n = 19)	UCD Group (n = 20)	TCD Group (n = 45)	Diagnosis (n = 13)	GFD 6 Months (n = 13)	GFD 12 months (n = 13)
Fecal pH	6.9 (0.08)	6.9 (0.15) [1]	6.7 (0.3)	7.1 (0.1) [2]	6.4 (0.46)	7.2 (0.18) [1]	6.8 (0.16) [1]
Fecal water content (*%*)	67.8 (0.7)	65.5 (1.6)	66.3 (1.7)	69.2 (1.2) [2]	67.6 (0.46)	65.8 (1.1)	69.1 (2.0)
Ammonia *(×10–*^*4*^*mg/g*)	11.5 (0.8)	11.4 (1.4)	19.6 (8.2)	7.8 (0.8)^a^ [2]	11.2 (0.96)	8.0 (1.2)	11.1 (1.6) [2]
Free sulfide (*μmol/g*)	0.13 (0.02) [2]	0.13 (0.03)	0.06 (0.01)^b^ [2]	0.10 (0.01) [4]	0.03 (0.01) [1]	0.05 (0.01)	0.09 (0.02)
Total sulfide (*μmol/g*)	0.83 (0.10)	1.15 (0.17)	0.83 (0.13) [2]	1.03 (0.11) [3]	0.87 (0.17) [1]	0.72 (0.13)	0.57 (0.11)
L-lactic acid (*μg/g*)	126.8 (41.3)	114.0 (11.1) [2]	60.1 (14.0)^c^^d^ [1]	100.8 (11.6) [7]	55.3 (16.6)	67.6 (10.9)	81.5 (11.2)
D-lactic acid (*μg/g*)	92.5 (18.4)	60.4 (6.4) [2]	119.1 (23.6) [1]	62.5 (4.1)^e^ [7]	130.2 (33.9)	116.9 (16.9)	123.2 (15.6)
Total lactic acid (*μg/g*)	219.3 (59.4)	174.4 (11.6) [2]	179.2 (29.0) [1]	163.3 (13.6) [7]	185.5 (38.6)	184.4 (17.5)	204.7 (18.0)
Acetic acid (*μmol/g*)	128.2 (5.2)	117.9 (8.6)	119.9 (10.0) [1]	124.6 (6.9) [2]	121.9 (13.8)	124.0 (12.6)	116.6 (11.1)
Propionic acid (*μmol/g*)	25.8 (1.6)	23.1 (3.0)	23.2 (2.7) [1]	23.1 (2.1) [2]	26.7 (3.5)	22.0 (2.9)	23.6 (3.0)
Butyric acid (*μmol/g*)	24.4 (1.5)	21.9 (2.8)	23.2 (3.3) [1]	21.0 (2.5) [2]	26.7 (4.4)	19.8 (5.4)	20.5 (2.5)
Valeric acid (*μmol*/*g*)	3.1 (0.18)	2.9 (0.31)	3.0 (0.37) [1]	2.5 (0.24) [2]	3.2 (0.51)	2.3 (0.37)	2.4 (0.33)
Isobutyric acid (*μmol/g*)	3.6 (0.22)	3.5 (0.35)	3.8 (0.46) [1]	3.0 (0.25) [2]	4.0 (0.64)	3.1 (0.37)	2.7 (0.35)
Isovaleric acid (*μmol/g*)	3.6 (0.23)	3.5 (0.32)	3.9 (0.48) [1]	3.1 (0.28) [2]	4.1 (0.66)	3.1 (0.37)	2.6 (0.33)
Total SCFAs (*μmol/g*)	188.6 (7.9)	172.8 (14.6)	176.9 (15.9)	177.3 (11.4)	186.6 (21.7)	174.4 (21.3)	168.5 (14.9)

NOTE. Values are mean (SEM). General linear models for UCD and TCD groups and HCs in the cross-sectional study, and general linear models accounted for paired data in the prospective study. Box-Cox transformation with optimal λ; pairwise comparison, Bonferroni method; the number of missing data is shown in brackets. Metabolites are measured per wet matter.

∗for all groups comparison

a *P* = .001 compared with HCs and UCD group.

b *P* = .009 compared with HCs.

c *P* = .012 compared with HCs.

d *P* = .003 compared with TCD group.

e *P* = .037 compared with UCD group.

In the prospective group of UCD patients the absolute concentrations of butyric acid (P = .053) and 2 BCFAs (isovaleric acid, P = .052; isobutyric acid, P = .063) nonsignificantly decreased after GFD initiation (Table 2). The effect of a GFD on SCFA production was reflected also in their proportional profile (Figure 5*B*). Compared with disease diagnosis, the relative abundance of acetic acid increased and the relative abundance of propionic, butyric, and valeric acids decreased at 6 and 12 months on a GFD, mirroring the observations in the cross-sectional cohort and between the TCD children and HCs or UCD children.

Samples from TCD children had lower ammonia concentration than HCs or UCD children, and patients with UCD had significantly less free sulfide than HCs (Table 2). Mean fecal L-lactic acid concentration was significantly lower in UCD than TCD children and HCs, but its D-isomer was higher in UCD than TCD children. During the follow-up of the 13 UCD children, a nonsignificant (P = .067) decrease in ammonia levels and a corresponding increase in free sulfide (P = .074) and L-lactate (P = .087) concentrations were observed. There was no difference in bacterial metabolites, except for fecal ammonia, which was significantly lower in patients who had undetectable GIP than those who had consumed gluten (Supplementary Table 6).

## Discussion

It is still unclear the extent to which an altered microbiota observed in previous research^8^, ^9^, ^10^, ^11^, ^12^, ^13^, ^14^, ^15^, ^16^, ^17^ is involved in CD pathogenesis or if these are secondary effects of disease pathology, including increased epithelial cell turnover and nutrient malabsorption. After diagnosis, adherence to a GFD may be associated with a decreased intake of nondigestible carbohydrates from cereals, thus affecting fiber-fermenting species and colonic production of SCFAs.^32^ This is important because CD patients may be unable to compensate for decreased fiber intake from gluten-containing foods by increasing its intake from other sources, including fruits and vegetables. Here, by performing a data-rich study, we tried to discern which microbial signals in patients with new-onset and treated CD are potentially involved in disease pathogenesis and which are secondary disease effects, including from treatment.

Even though we identified differences in the abundance of a few species between UCD patients and HCs, the profound microbial dysbiosis noted in Crohn’s disease was not observed, at least using crude diversity indices.^18^ Instead, significant effects were observed in TCD patients after recommendation to a GFD, confirming our a priori hypothesis. More importantly, we identified 3 major groups of bacterial taxa (Figure 2): 1 group that is CD-specific and nonresponsive to treatment with GFD, a second group associated with new-onset CD but is also treatment responsive, and a third group that is treatment dependent but does not differentiate between disease and the health state. Of these, the first cluster represents the microbial signature of CD that can distinguish children with from those without CD with a reasonably high likelihood, as demonstrated using machine learning algorithms. The magnitude of microbial alterations observed here are similar to other noncommunicable diseases in which the microbiota has been implicated in their underlying pathogenesis, such as type 1 diabetes.^33^

Although in the second group several other bacteria were different between UCD children and the HCs, these discriminatory microbial signals vanished after treatment with a GFD. These represent bacteria that responded to recovery of gut pathology after treatment with a GFD or bacteria whose underlying role in CD cause and treatment might be important. The role of these taxa, most of which belong to Bacteroidetes, warrants further research. Using denaturing gradient gel electrophoresis with group-specific primers, Sánchez et al^15^ also showed that *Bacteroides* diversity was higher in duodenal biopsies from control subjects than in samples from patients with active and treated CD.

**Supplementary Figure 5:**
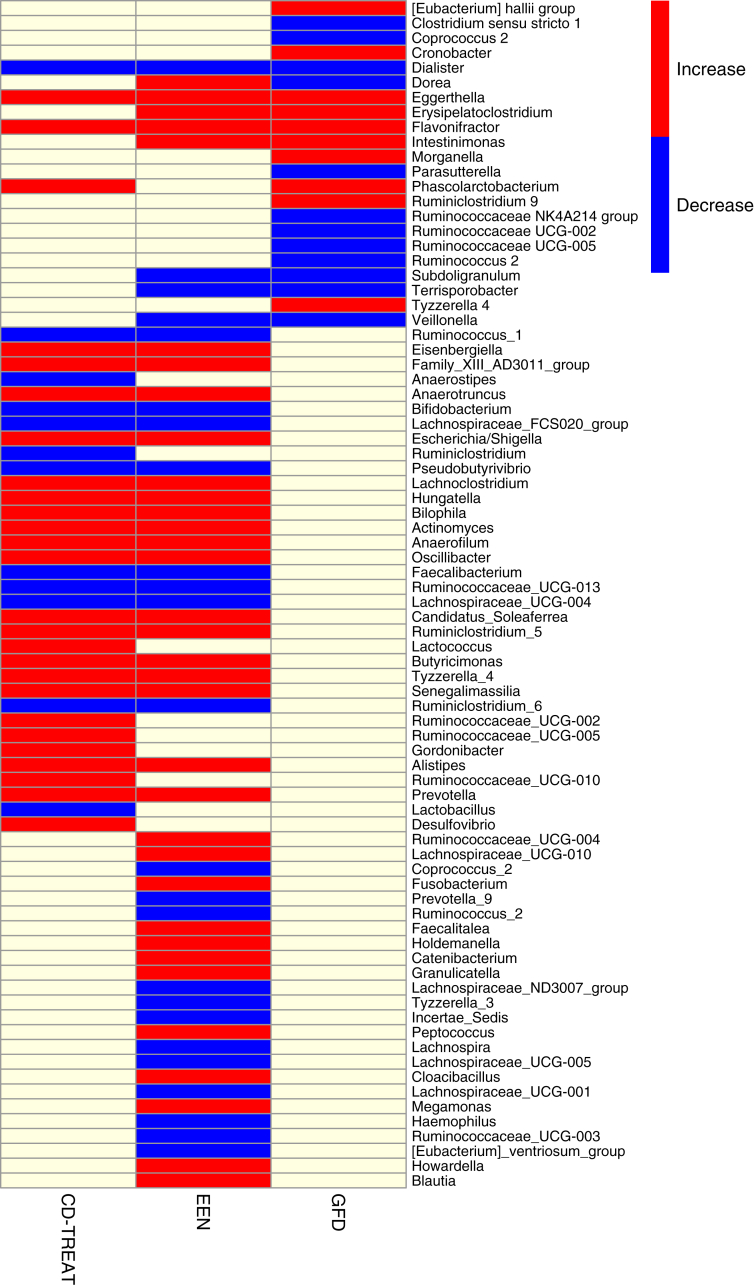
Changes in genera during a GFD in children with CD in the current study and their overlap with genera that changed after treatment with exclusive enteral nutrition (EEN) or a new dietary therapy for Crohn’s disease (CD-TREAT) in healthy adults from previous research.^25^*Red* indicates increase and *blue* decrease in relative abundance.

The third and largest group should almost certainly represent microbial noise attributed to dietary modification during treatment with a GFD and amelioration of disease activity. This speculation is supported by the fact that several differential OTUs between the TCD with the UCD children and HCs overlapped; most of these discriminatory species were associated with participants’ diet and by the observation that in patients on a GFD almost 100 OTUs differed between patients with positive and negative gluten contents in their feces. As a prime example of these effects, avoidance of wheat products likely explains the decrease of *Megamonas* in TCD patients,^34^ and the same may apply for the fiber fermenters *Coprococcus*, *Ruminococcus* and *Anaerostipes,* and *Bifidobacterium*. The reduction in *Bifidobacterium* in CD is in accordance with previous research using molecular fingerprinting techniques^17^ and quantitative polymerase chain reaction.^9^ Of note, 41% of the genera that were influenced by a GFD in the current study were also influenced in healthy adults who complied with other dietary interventions that were gluten free and low in fiber (Supplementary Figure 5) in previous research.^25^ This further corroborates our conclusions that to a large extent OTUs that are altered in TCD are the result of a GFD and low fiber intake. Changes in the abundance of butyric acid producers paralleled a decrease in butyric acid levels and its proportional abundance in patients on a GFD. This finding confirms previous observations,^12^ but here we provide evidence that this is a secondary effect and not a primary disease defect. The concentration of ammonia and BCFA was lower in patients on a GFD, both in the cross-sectional and prospective cohorts. This likely indicates reduction in protein intake or lower epithelial cell shedding, with amelioration of intestinal inflammation, both resulting in less protein reaching the colon and being fermented. The reason behind the low concentration of free sulfide at disease diagnosis is unclear but in conjunction with the significantly reduced abundance of Methanobrevibacter, a methane producer, suggests an altered hydrogen metabolism in newly diagnosed CD. Hydrogen sulfide has been implicated in various processes of gut function, including motility, epithelial secretion, and protection from inflammation.^35^

Findings from the prospective cohort corroborated the results of the cross-sectional group analysis, although often different species were affected by GFD between the 2 cohorts, highlighting substantial variation in interindividual responses. The observation that almost 3 times fewer species were affected after the 12-month compared with the 6-month diagnosis indicates either better adaptation of CD patients to a GFD and broader food choices to compensate for gluten-containing food with time or, most likely and supported by the change in GIP levels between these periods, loss of strict adherence to a GFD in some patients.

There are significant implications for future research and clinical practice arising from the findings of this study. The role of CD-associated microbiota in disease pathogenesis, including those organisms that respond to a GFD, needs to be unraveled in mechanistic research. The fact that the CD microbial signature we observed persisted in patients on a GFD and was independent of disease duration suggests that the effects on these 11 OTUs are unrelated to bacterial fermentation of the luminal glycocalyx and other GI secretions or mediators of the innate immune system. It is therefore possible that these bacterial species are important modifiers of risk of CD onset, particularly in individuals who are genetically susceptible to developing the illness. In previous research, healthy exclusively breastfed infants who were carriers of the HLA-DQ2 haplotype with a family history of CD had less Bifidobacterium.^36^ Provided that most patients with CD are carriers of the HLA-DQ2 haplotype, the observation of a lower Bifidobacterium abundance reported in the current and previous studies^36^ suggests that genetic factors that impede early colonization of the gut with species beneficial for human health may potentiate the risk of development of CD; this effect extends beyond disease diagnosis and remains independent of GFD treatment.

The role of Bifidobacterium in the underlying microbial origins of CD pathogenesis has received extensive attention within mechanistic research. Inoculation of peripheral blood mononuclear cells with feces from active and asymptomatic CD patients increased tumor necrosis factor-α production and CD86 expression and decreased interleukin-10 cytokine production and CD4 expression compared with samples from HCs, but specific Bifidobacterium strains suppressed this helper T cell type 1 proinflammatory milieu, characteristic of CD.^37^ In a subsequent study of the same group, addition of Bifidobacterium strains changed the gliadin-derived peptide pattern and attenuated production of tumor necrosis factor-α and interleukin-1β and expression of NF-κB and chemokine CXCR3 receptor from Caco-2 cells exposed to gliadin digestions.^38^ These in vitro data were replicated in gliadin-induced enteropathy murine models sensitized with interferon-γ where Bifidobacterium longum CECT 7347 attenuated the production of tumor necrosis factor-α and the CD4 mediated immune response and increased the tissue messenger NA levels of NF-κB and interleukin-10.^39^

The exact mechanism by which bifidobacteria may exhibit immune-modulating properties is not yet clear, but it has been demonstrated that Bifidobacterium longum NCC2705 produces a serine protease inhibitor that attenuates gliadin-induced immunopathology and impacts intestinal microbial composition in the NOD/DQ8 mouse model of gluten sensitivity.^40^ In 1 of the few clinical trials available, administration of Bifidobacterium infantis decreased Paneth cells and expression of α-defensin-5 in duodenal biopsies of patients with active CD,^41^ an effect that was associated with symptom improvement but did not modify abnormal intestinal permeability.^42^

Very few other species identified here as disease-specific biomarkers have been studied in the context of CD pathogenesis. Commensal Clostridia belonging to clusters IV and XIVa are important inducers of regulatory T cells in the colon.^43^ It is therefore possible that the highly discriminant OTU_53 belonging to an unknown Clostridium is less abundant in CD, thus influencing the induction of regulatory T cells required for maintaining immune homeostasis. It has also been shown that bacteria could potentially reduce gluten immunogenicity by producing enzymes that effectively cleave proteolytic-resistant sequences in gluten peptides that activate helper T cell type 1 response.^44^ Pseudomonas aeruginosa, isolated from the duodenum of CD patients, produces, through its elastase activity, a multitude of peptides that activate gluten-specific T cells in HLA-DQ2 CD patients, but conversely Lactobacillus spp from healthy subjects degrade such modified peptides and decrease their immunogenic potential.^45^

Future research should explore the role of the disease-specific species identified here in disease pathogenesis. Such studies may include in vitro experiments with candidate species and immune cell co-cultures triggered by gliadin epitopes and dietary interventions aiming to change their abundance in the gut coupled with measurements of disease outcomes.^13^ The ability of microbiota signatures of unaffected siblings to predict the risk of CD onset alongside other environmental factors is important to study and in a similar way to ongoing large cohort studies in Crohn’s disease.^46^

Irrespective of their primary role in CD pathogenesis, the disease-specific microbial signature identified here might be used as another adjutant, noninvasive biomarker to screen for CD. The observation that the abundance of fiber fermenters or cross-feeders and production of butyric acid diminish in patients on a GFD has implications for the dietary management of this population. Dietary fiber intake in the westernized diet is low, and adherence to a GFD with low consumption of cereals may decrease patient intake even further. It is therefore important for colonic health and gut motility to promote intake of nongluten-containing sources of fiber in this population and routinely fortify gluten-free products with a broad variety of fibers (eg, pectin, ispaghula).

Limitations of the current study include the modest sample size of the prospective group. Although the mean effect size of microbial changes was more pronounced than the cross-sectional group, we may have been underpowered to identify smaller size differences. Some patients from the UCD group were lost at follow-up or their measurements were excluded. However, this group of patients did not differ in characteristics and microbiota features from patients who were retained in the analysis. Also, CD is a condition of the small bowel; hence, the role of fecal microbiota may be considered less relevant to its pathogenesis. Although this is a fair argument to propose, it is possible that events in the large bowel influence disease pathogenesis upstream along the GI tract, as is perhaps the case in Crohn’s disease where colonic microbiota changes can be seen in patients with disease affecting their small intestine. It is also possible that several fecal microbes are markers of the small bowel resident community. Collado et al^47^ previously showed that similar bacteria were related to CD in both fecal and duodenal biopsies, but further research is required to clarify the role of each of these gut niches and in the mucosal-adherent microbiota as suggested.^9^,^48^ In HCs we did not have ethical permission to screen for CD. However, that none of the siblings of the CD patients screened positive for CD infers that the proportion of HCs with undiagnosed CD would have been small and unlikely to have influenced the main results presented here.

In conclusion, we identified a set of bacteria that may comprise another important environmental factor in the pathogenesis of CD and that warrant further research. We also demonstrated that several alterations in the microbiota of patients with established CD are likely to be secondary effects of disease treatment. The suppression of butyric acid production and fiber fermenters is likely a biomarker of diminished consumption of fermentable carbohydrate and may suggest a need for the development of fiber-enriched, gluten-free products and interventions with prebiotics.

## Figures and Tables

**Supplementary Table 1:** PERMANOVA Analysis Using Bray-Curtis and Unweighted Unifrac Distance Matrices at OTU and Genus Levels

Levels	Group	Bray-Curtis	Unweighted UniFrac
*OTU level*			
Overall		*P* = .025, *R*^2^ = 2.76%	*P* = .027, *R*^2^ = 2.49%
Within-group comparison			
UCD (20)	HCs (57)	*P* = .506, *R*^2^= 1.21%	*P* = .125, *R*^2^ = 1.77%
TCD (45)	HCs (57)	*P* = .017, *R*^2^ = 2.32%	*P* = .045, *R*^2^ = 1.57%
	UCD (20)	*P* = .106, *R*^2^ = 2.35%	*P* = .056, *R*^2^ = 2.46%
UCD (13)	6-mo GFD (13)	*P* = .951, *R*^2^ = 1.53%	*P* = .377, *R*^2^ = 2.54%
	12-mo GFD (13)	*P* = .762, *R*^2^ = 2.42%	*P* = .691, *R*^2^ = 2.44%
Siblings (19)	TCD (18)	*P* = .384, *R*^2^ = 2.89%	*P* = .336, *R*^2^ = 2.96%
	HCs (57)	*P* = .745, *R*^2^ = 0.97%	*P* = .183, *R*^2^ = 1.67%
*Genus level*			
Overall		*P* = .026, *R*^2^ = 3.0%	n/a
Within-group comparison			
UCD (20)	HCs (57)	*P* = .515, *R*^2^ = 1.15%	n/a
TCD (45)	HCs (57)	*P* = .013, *R*^2^ = 2.56%	n/a
	UCD (20)	*P* = .069, *R*^2^ = 2.77%	n/a
UCD (13)	6-mo GFD (13)	*P* = .998, *R*^2^ = 1.28%	n/a
	12-mo GFD (13)	*P* = .904, *R*^2^ = 2.21%	n/a
Siblings (19)	TCD (18)	*P* = .296, *R*^2^ = 3.21%	n/a
	HCs (57)	*P* = .696, *R*^2^ = 0.94%	n/a

NOTE. n/a, not applicable; PERMANOVA, permutational multivariate analysis of variance.

**Supplementary Table 2:** Bioenv Selected OTU That Explain Part of the Variance in Microbiota Structure Described by the Full OTU Dataset

**92.3% in UCD, TCD and HC**
“OTU_6:Bacteria;Firmicutes;Negativicutes;Selenomonadales;Veillonellaceae;*Dialister*;”
“OTU_4:Bacteria;Bacteroidetes;Bacteroidia;Bacteroidales;Bacteroidaceae;*Bacteroides*;”
“OTU_2:Bacteria;Actinobacteria;Actinobacteria;Bifidobacteriales;Bifidobacteriaceae;*Bifidobacterium*;”
“OTU_881:Bacteria;Firmicutes;Clostridia;Clostridiales;Ruminococcaceae;*Faecalibacterium*;”
“OTU_14:Bacteria;Firmicutes;Clostridia;Clostridiales;Ruminococcaceae;*Subdoligranulum*;”
“OTU_33:Bacteria;Firmicutes;Clostridia;Clostridiales;Lachnospiraceae;*Roseburia*;”
“OTU_129:Bacteria;Actinobacteria;Coriobacteriia;Coriobacteriales;Coriobacteriaceae;*Eggerthella*;”
“OTU_568:Bacteria;Actinobacteria;Actinobacteria;Bifidobacteriales;Bifidobacteriaceae;*Bifidobacterium;Bifidobacterium bifidum NCIMB 41171”*
“OTU_72:Bacteria;Firmicutes;Clostridia;Clostridiales;Lachnospiraceae;*Lachnoclostridium*;”
“OTU_655:Bacteria;Bacteroidetes;Bacteroidia;Bacteroidales;Bacteroidaceae;*Bacteroides*;”
“OTU_35:Bacteria;Firmicutes;Clostridia;Clostridiales;Lachnospiraceae;*Blautia*;”
“OTU_20:Bacteria;Bacteroidetes;Bacteroidia;Bacteroidales;Bacteroidaceae;*Bacteroides*;”
“OTU_458:Bacteria;Firmicutes;Clostridia;Clostridiales;Lachnospiraceae;*Lachnospiraceae UCG-008*;”
**92% in UCD and TCD**
“OTU_6∗:Bacteria;Firmicutes;Negativicutes;Selenomonadales;Veillonellaceae;*Dialister*;”
“OTU_4∗:Bacteria;Bacteroidetes;Bacteroidia;Bacteroidales;Bacteroidaceae;*Bacteroides*;”
“OTU_2∗:Bacteria;Actinobacteria;Actinobacteria;Bifidobacteriales;Bifidobacteriaceae;*Bifidobacterium*;”
“OTU_881:Bacteria;Firmicutes;Clostridia;Clostridiales;Ruminococcaceae;*Faecalibacterium*;”
“OTU_60∗:Bacteria;Bacteroidetes;Bacteroidia;Bacteroidales;Bacteroidaceae;*Bacteroides*;”
“OTU_14∗:Bacteria;Firmicutes;Clostridia;Clostridiales;Ruminococcaceae;*Subdoligranulum*;”
“OTU_33∗:Bacteria;Firmicutes;Clostridia;Clostridiales;Lachnospiraceae;*Roseburia*;”
“OTU_129∗:Bacteria;Actinobacteria;Coriobacteriia;Coriobacteriales;Coriobacteriaceae;*Eggerthella*;”
“OTU_568∗:Bacteria;Actinobacteria;Actinobacteria;Bifidobacteriales;Bifidobacteriaceae;*Bifidobacterium;Bifidobacterium bifidum NCIMB 41171”*
“OTU_72∗:Bacteria;Firmicutes;Clostridia;Clostridiales;Lachnospiraceae;*Lachnoclostridium*;”
“OTU_35:Bacteria;Firmicutes;Clostridia;Clostridiales;Lachnospiraceae;*Blautia*;”
“OTU_20:Bacteria;Bacteroidetes;Bacteroidia;Bacteroidales;Bacteroidaceae;*Bacteroides*;”
“OTU_10:Bacteria;Firmicutes;Clostridia;Clostridiales;Ruminococcaceae;*Faecalibacterium*;”
“OTU_57:Bacteria;Firmicutes;Clostridia;Clostridiales;Lachnospiraceae;*Lachnospira*;”
**91.4% in HC and TCD children**
“OTU_6∗:Bacteria;Firmicutes;Negativicutes;Selenomonadales;Veillonellaceae;*Dialister*;”
“OTU_4∗:Bacteria;Bacteroidetes;Bacteroidia;Bacteroidales;Bacteroidaceae;*Bacteroides*;”
“OTU_2∗:Bacteria;Actinobacteria;Actinobacteria;Bifidobacteriales;Bifidobacteriaceae;*Bifidobacterium*;”
“OTU_60∗:Bacteria;Bacteroidetes;Bacteroidia;Bacteroidales;Bacteroidaceae;*Bacteroides*;”
“OTU_14∗:Bacteria;Firmicutes;Clostridia;Clostridiales;Ruminococcaceae;*Subdoligranulum*;”
“OTU_33∗:Bacteria;Firmicutes;Clostridia;Clostridiales;Lachnospiraceae;*Roseburia*;”
“OTU_129∗:Bacteria;Actinobacteria;Coriobacteriia;Coriobacteriales;Coriobacteriaceae;*Eggerthella*;”
“OTU_568∗:Bacteria;Actinobacteria;Actinobacteria;Bifidobacteriales;Bifidobacteriaceae;Bifidobacterium;*Bifidobacterium bifidum NCIMB 41171”*
“OTU_72∗:Bacteria;Firmicutes;Clostridia;Clostridiales;Lachnospiraceae;*Lachnoclostridium;”*
“OTU_655:Bacteria;Bacteroidetes;Bacteroidia;Bacteroidales;Bacteroidaceae;*Bacteroides;”*
“OTU_86:Bacteria;Bacteroidetes;Bacteroidia;Bacteroidales;Rikenellaceae;*Alistipes;”*
“OTU_41:Bacteria;Firmicutes;Clostridia;Clostridiales;Peptostreptococcaceae;*Intestinibacter;”*

NOTE. ∗Asterisk indicates Bioenv-selected OTUs that partially explain the variance in community structure of UCD vs TCD as well as HC vs TCD; OTU: operational taxonomic unit

**Supplementary Table 3:** OTUs With Significantly Different Relative Abundance in Fecal Samples of UCD and TCD Children and HCs

	Group Comparison	Base Mean	Log2-fold Change	*P*	*P* (Adjusted)
OTU_60 *Bacteroides*	HC vs UCD	7.06	–0.23	6.3E-01	7.83E-01
	HC vs TCD	122.96	5.17	3.03E-23	2.17E-20
	UCD vs TCD	80.26	4.15	1.43E-08	8.46E-07
OTU_143 *Ruminococcus* 1	HC vs UCD	25.1	–2.08	8.85E-04	1.16E-02
	HC vs TCD	18.11	–4.37	8.31E-23	2.98E-20
	UCD vs TCD	3.30	–2.28	5.05E-08	2.44E-06
OTU_31 *Megamonas*	HC vs UCD	39.6	–2.09	4.56E-03	3.55E-02
	HC vs TCD	28.46	–4.72	3.43E-21	8.21E-19
	UCD vs TCD	4.94	–2.64	3.99E-08	2.12E-06
OTU_244 *Ruminococcus* 1	HC vs UCD	24.72	–1.37	3.2E-02	1.06E-01
	HC vs TCD	17.06	–4.26	5.95E-21	1.07E-18
	UCD vs TCD	2.15	–1.19	1.28E-03	9.45E-03
OTU_78 *Ruminiclostridium* 5	HC vs UCD	2.71	–0.69	9.89E-02	2.34E-01
	HC vs TCD	30.16	4.40	1.54E-20	2.21E-18
	UCD vs TCD	44.97	5.09	1.97E-12	5.22E-10
OTU_42 *Methanobrevibacter*	HC vs UCD	38.78	–3.84	1.33E-07	1.47E-05
	HC vs TCD	29.62	–4.36	1.88E-17	2.24E-15
	UCD vs TCD	2.84	–0.51	2.78E-01	4.25E-01
OTU_114 *Caprococcus* 2	HC vs UCD	32.92	–0.25	7.13E-01	8.41E-01
	HC vs TCD	38.73	–4.27	2.92E-17	2.99E-15
	UCD vs TCD	4.24	–0.79	1.22E-01	2.32E-01
OTU_70 *Ruminicoccaceae*	HC vs UCD	11.29	–3.43	1.28E-07	1.47E-05
UCG-014	HC vs TCD	8.89	–3.36	5.94E-14	5.32E-12
	UCD vs TCD	1.42	0.07	8.52E-01	n/a
OTU_120 *Lachnospiraceae*	HC vs UCD	4.48	0.63	1.34E-01	2.97E-01
UCG-005	HC vs TCD	69.03	3.59	7.21E-13	5.74E-11
	UCD vs TCD	99.05	4.43	3.74E-11	3.43E-09
OTU_135 *Holdemanella*	HC vs UCD	21.19	–1.04	1.10E-01	2.55E-01
	HC vs TCD	14.82	–3.20	4.31E-12	3.09E-10
	UCD vs TCD	5.52	–2.20	3.0E-06	7.23E-05
OTU_197 *Ruminococcaceae*	HC vs UCD	8.55	1.18	1.27E-02	5.63E-02
UCG-005	HC vs TCD	22.96	2.76	1.12E-11	7.29E-10
	UCD vs TCD	23.17	0.85	1.42E-01	2.56E-01
OTU_908 *Anaerostipes*	HC vs UCD	12.70	–0.83	1.60E-01	3.32E-01
	HC vs TCD	8.98	–2.66	4.46E-10	2.66E-08
	UCD vs TCD	4.04	–1.83	2.57E-04	2.73-03
OTU_979 *Bifidobacterium*	HC vs UCD	45.70	–0.18	7.36E-01	8.59E-01
*Pseudocatenulatum*	HC vs TCD	31.04	–2.13	5.39E-09	2.74E-07
	UCD vs TCD	19.91	–1.97	3.0E-05	5.69E-04
OTU_53 *Clostridium*	HC vs UCD	200.98	–2.58	1.1E-05	4.04E-04
sensu stricto 1	HC vs TCD	239.45	–2.77	5.72E-09	2.74E-07
	UCD vs TCD	52.52	0.39	5.09E-01	6.45E-01
OTU_186	HC vs UCD	1.35	–0.11	7.66E-01	n/a
*Gastranaerophilales*	HC vs TCD	3.42	2.11	5.74E-09	2.74E-07
	UCD vs TCD	4.53	2.22	7.91E-05	1.14E-03
OTU_84 *Enterorhabdus*	HC vs UCD	7.93	–2.34	1.20E-04	2.54E-03
	HC vs TCD	6.52	–2.32	3.29E-08	1.48E-06
	UCD vs TCD	1.99	0.02	9.53E-01	9.71E-01
OTU_174 *Catenibacterium*	HC vs UCD	1.56	0.70	2.74E-02	n/a
	HC vs TCD	3.19	2.04	3.64E-08	1.54E-06
	UCD vs TCD	4.47	1.32	1.81E-02	7.2E-02
OTU_336 *Holdemanella*	HC vs UCD	7.99	–2.38	6.68E-05	1.76E-03
	HC vs TCD	6.59	–2.30	6.27E-08	2.5E-06
	UCD vs TCD	2.02	0.06	8.75E-01	9.27E-01
OTU_776 *Bifidobacterium*	HC vs UCD	21.81	–2.25	5.29E-04	8.59E-03
*Animalis*	HC vs TCD	17.47	–2.50	9.08E-08	3.26E-06
	UCD vs TCD	4.47	1.32	1.81E-02	7.2E-02
OTU_88 *Bacteroides*	HC vs UCD	281.31	0.02	9.79E-01	9.82E-01
	HC vs TCD	863.13	2.49	1.02E-07	3.48E-06
	UCD vs TCD	1,187.7	2.48	2.66E-04	2.77E-03
OTU_1002 *Lachnospira*	HC vs UCD	8.97	–1.75	8.59E-04	1.16E-02
	HC vs TCD	33.92	2.49	1.08E-07	3.51E-06
	UCD vs TCD	45.22	3.23	1.54E-06	3.89E-05
OTU_28 *Bacteroides*	HC vs UCD	151.07	–1.06	6.32E-02	1.7E-01
	HC vs TCD	728.21	2.57	2.80E-07	8.72E-06
	UCD vs TCD	552.60	3.13	1.08E-06	2.85E-05
OTU_1049 *Lachnoclostridium*	HC vs UCD	53.35	0.60	3.14E-01	5.04E-01
	HC vs TCD	128.17	2.28	8.26E-07	2.28E-05
	UCD vs TCD	180.41	1.68	1.09E-02	5.23E-02
OTU_125 *Parabacteroides*	HC vs UCD	34.72	–1.85	9.65E-04	1.22E-02
	HC vs TCD	116.40	2.28	9.30E-07	2.47E-05
	UCD vs TCD	148.10	4.02	3.88E-11	3.43E-09
OTU_278 *Akkermansia*	HC vs UCD	4.46	0.34	4.50E-01	6.47E-01
	HC vs TCD	15.59	2.17	1.13E-06	2.88E-05
	UCD vs TCD	20.63	2.37	1.87E-04	2.2E-03
OTU_537 *Dialister*	HC vs UCD	18.0	–2.46	1.10E-04	2.43E-03
	HC vs TCD	15.10	–2.20	1.37E-06	3.40E-05
	UCD vs TCD	4.68	0.23	6.11E-01	7.29E-01
OTU_62 *Ruminococcaceae*	HC vs UCD	23.89	–3.94	1.84E-08	1.01E-05
UCG-014	HC vs TCD	11.97	–2.27	1.69E-06	3.90E-05
	UCD vs TCD	3.30	0.88	7.0E-02	1.79E-01
OTU_752 *Subdoligranulum*	HC vs UCD	43.81	–1.87	1.49E-03	1.68E-02
	HC vs TCD	164.0	2.47	2.72E-06	5.57E-05
	UCD vs TCD	122.66	3.48	5.24E-07	1.74E-05
OTU_99 *Senegalimassilia*	HC vs UCD	73.24	–3.88	1.05E-07	1.47E-05
	HC vs TCD	61.70	–2.47	9.10E-06	1.52E-04
	UCD vs TCD	14.06	1.48	2.37E-02	8.5E-02
OTU_112 *Bacteroidetes*	HC vs UCD	22.60	–3.07	1.16E-07	1.47E-05
	HC vs TCD	31.79	–1.19	1.0E-02	4.2E-02
	UCD vs TCD	13.92	2.42	1.43E-05	3.15E-04
OTU_1054 *Alistipes*	HC vs UCD	17.29	–2.95	7.37E-07	6.78E-05
	HC vs TCD	30.12	0.81	8.83E-02	2.18E-01
	UCD vs TCD	28.41	3.74	1.82E-09	1.38E-07
OTU_146 *Slackia*	HC vs UCD	7.95	–3.09	1.02E-06	8.05E-05
	HC vs TCD	7.16	–1.73	1.27E-04	1.26E-03
	UCD vs TCD	2.57	1.36	9.44E-03	4.91E-02
OTU_226 [*Eubacterium*]	HC vs UCD	5.92	–2.79	2.55E-06	1.76E-04
*oxideroducens* group	HC vs TCD	6.68	–0.48	2.6E-01	4.48E-01
	UCD vs TCD	4.11	2.37	4.87E-05	8.33E-04
OTU_45 *Parabacteroides*	HC vs UCD	56.41	–3.08	3.60E-06	2.21E-04
	HC vs TCD	73.21	0.84	1.04E-01	2.47E-01
	UCD vs TCD	69.9	3.44	3.06E-07	1.25E-05
OTU_895 *Barnesiella*	HC vs UCD	58.0	–3.11	5.04E-06	2.77E-04
	HC vs TCD	54.13	–1.48	5.16E-03	2.4E-02
	UCD vs TCD	21.56	1.65	1.02E-02	5.03E-02
OTU_101 *Anaerotruncus*	HC vs UCD	15.61	–3.11	5.53E-06	2.77E-04
	HC vs TCD	17.95	–0.46	3.83E-01	5.67E-01
	UCD vs TCD	11.03	2.64	4.55E-05	8.05E-04
OTU_572 Family XVIII	HC vs UCD	9.54	–2.43	7.14E-06	3.16E-04
AD3011 group	HC vs TCD	11.51	–0.19	6.55E-01	7.82E-01
	UCD vs TCD	7.94	2.20	6.21E-05	9.70E-04
OTU_230 *Bacteroides*	HC vs UCD	6.20	–2.79	7.43E-06	3.16E-04
	HC vs TCD	6.79	–0.60	1.79E-01	3.62E-01
	UCD vs TCD	4.02	2.18	1.13E-04	1.58E-03
OTU_65 *Bacteroides*	HC vs UCD	9.79	–2.44	8.18E-06	3.23E-04
	HC vs TCD	25.82	1.73	7.37E-05	1.28E-03
	UCD vs TCD	29.9	4.19	1.17E-12	5.22E-10
OTU_49 *Alistipes*	HC vs UCD	56.57	–2.40	1.86E-05	6.42E-04
	HC vs TCD	174.05	1.50	3.31E-03	1.72E-02
	UCD vs TCD	192.53	4.41	5.95E-12	1.05E-09
OTU_109 *Ruminococcus* 2	HC vs UCD	6.0	–2.54	2.39E-05	7.75E-04
	HC vs TCD	5.40	–1.57	2.12E-04	1.88E-03
	UCD vs TCD	2.22	0.96	3.83E-02	1.17E-01
OTU_51 *Ruminococcaceae*	HC vs UCD	13.48	–2.49	4.88E-05	1.50E-03
UCG-014	HC vs TCD	16.15	–0.22	6.38E-01	7.66E-01
	UCD vs TCD	11.22	2.25	1.20E-04	1.60E-03
OTU_111 *Blautia*	HC vs UCD	23.54	–2.33	6.14E-05	1.7E-03
	HC vs TCD	28.78	–0.13	7.87E-01	8.72E-01
	UCD vs TCD	20.82	2.19	4.69E-04	4.61E-03
OTU_868 *Bacteroides*	HC vs UCD	136.18	–2.64	7.33E-05	1.76E-03
	HC vs TCD	293.71	1.33	1.42E-02	5.4E-02
	UCD vs TCD	310.64	4.83	8.47E-12	1.12E-09
OTU_923 *Mavinbryantia*	HC vs UCD	10.23	–2.00	7.03E-05	1.76E-03
	HC vs TCD	13.06	0.07	8.63E-01	9.2E-01
	UCD vs TCD	10.29	2.06	2.49E-04	2.7E-03
OTU_190 *Alistipes*	HC vs UCD	6.26	–2.16	1.09E-04	2.43E-03
	HC vs TCD	11.02	0.07	8.74E-01	9.26E-01
	UCD vs TCD	13.20	3.35	5.57E-08	2.46E-06
OTU_168 *Alistipes*	HC vs UCD	4.43	–2.01	4.11E-04	7.31E-03
	HC vs TCD	3.92	–1.54	8.38E-05	9.40E-04
	UCD vs TCD	1.75	0.45	2.5E-02	3.9E-01
OTU_3 *Akkermansia*	HC vs UCD	2,041.4	–2.17	1.41E-03	1.63E-02
	HC vs TCD	2,863.9	0.33	5.55E-01	7.09E-01
	UCD vs TCD	2,466.0	1.45	5.12E-02	1.43E-01
OTU_1 *Prevotella* 9	HC vs UCD	6,760.4	–2.68	3.77E-03	3.15E-02
	HC vs TCD	5,953.8	–1.79	1.22E-02	4.84E-03
	UCD vs TCD	193.2	0.85	2.36E-01	3.75E-01
OTU_302 *Eisenbergiella*	HC vs UCD	26.32	–1.38	1.01E-02	4.74E-02
	HC vs TCD	55.97	1.44	7.21E-04	5.23E-03
	UCD vs TCD	97.89	3.49	2.47E-09	1.64E-07
OTU_259	HC vs UCD	4.86	–1.71	6.88E-04	1.07E-02
*Erysipelatoclostridium*	HC vs TCD	8.45	0.95	1.27E-02	5.0E-02
	UCD vs TCD	8.6	2.61	4.31E-07	1.64E-05
OTU_448 *Ruminiclostridium* 5	HC vs UCD	9.97	–1.66	5.36E-05	1.56E-03
	HC vs TCD	15.06	0.61	5.6E-02	1.57E-01
	UCD vs TCD	13.72	2.20	4.8E-07	1.7E-05
OTU_270 *Butyricimonas*	HC vs UCD	5.25	–1.88	4.56E-04	7.63E-03
	HC vs TCD	10.0	1.13	8.16E-03	3.52E-02
	UCD vs TCD	10.47	3.00	7.49E-07	2.34E-05
OTU_248 *Clostridium*	HC vs UCD	2.07	–0.77	7.32E-02	1.87E-01
sensu stricto 1	HC vs TCD	4.37	1.57	2.49E-05	3.5E-04
	UCD vs TCD	7.89	2.96	9.12E-07	2.69E-05
OTU_317 *Lachnospireaceae*	HC vs UCD	3.81	–1.83	1.52E-04	2.89E-03
*NK4A136* group	HC vs TCD	7.29	1.56	4.05E-03	1.99E-02
	UCD vs TCD	7.67	2.97	9.99E-07	2.79E-05
OTU_34 *Bacteroides*	HC vs UCD	243.37	–1.75	4.11E-03	3.29E-02
	HC vs TCD	425.59	0.96	3.8E-02	1.19E-01
	UCD vs TCD	431.67	2.72	6.24E-06	1.44E-04
OTU_235 *Lachnospiraceae*	HC vs UCD	6.69	–1.52	1.35E-03	1.60E-02
	HC vs TCD	10.74	0.79	3.9E-02	1.21E-01
	UCD vs TCD	10.62	2.31	1.95E-05	4.15E-04
OTU_1036 *Bacteroidetes*	HC vs UCD	59.31	–1.43	3.60E-02	1.14E-01
	HC vs TCD	76.45	0.23	6.5E-01	7.8E-01
	UCD vs TCD	61.94	2.57	2.07E-05	4.22E-04
OTU_73 *Bacteroides*	HC vs UCD	6.31	–1.26	8.05E-03	4.38E-02
	HC vs TCD	11.31	1.08	7.24E-03	3.24E-02
	UCD vs TCD	12.07	2.32	3.22E-05	5.89E-04
OTU_154 *Odoribacter*	HC vs UCD	30.05	–0.95	1.06E-01	2.48E-01
	HC vs TCD	61.85	1.48	1.13E-03	7.18E-03
	UCD vs TCD	72.08	2.43	5.23E-05	8.68E-04
OTU_139 *Bilophila*	HC vs UCD	22.05	–0.67	2.20E-01	3.99E-01
	HC vs TCD	49.71	0.44	9.57E-05	1.01E-03
	UCD vs TCD	59.99	2.37	5.65E-05	9.09E-04
OTU_77 *Lachnoclostridium*	HC vs UCD	47.57	–0.75	1.61E-01	3.32E-01
	HC vs TCD	120.45	1.11	1.82E-02	6.59E-02
	UCD vs TCD	128.1	2.40	7.94E-05	1.14E-03
OTU_343 *Christensenellaceae*	HC vs UCD	1.84	–0.42	2.68E-01	4.55E-01
	HC vs TCD	4.18	1.82	4.55E-07	1.36E-05
	UCD vs TCD	4.98	2.14	7.97E-05	1.14E-03
OTU_355 *Lachnoclostridium*	HC vs UCD	24.25	–0.88	9.18E-02	2.20E-01
	HC vs TCD	43.33	1.19	3.82E-03	1.9E-02
	UCD vs TCD	46.44	2.01	1.19E-04	1.60E-03
OTU_98 *Ruminococcaceae*	HC vs UCD	58.58	–0.55	3.0E-01	4.9E-01
	HC vs TCD	118.5	1.54	1.46E-04	1.36E-03
	UCD vs TCD	139.21	2.06	1.39E-04	1.80E-03
OTU_282 *Christensenellaceae*	HC vs UCD	4.57	–0.49	3.34E-01	5.24E-01
	HC vs TCD	11.23	1.37	1.8E-03	1.1E-02
	UCD vs TCD	12.46	2.21	1.59E-04	1.92E-03
OTU_1104 *Akkermansia*	HC vs UCD	8.04	–0.82	9.76E-02	2.32E-01
	HC vs TCD	17.76	1.65	2.25E-04	1.97E-03
	UCD vs TCD	21.41	2.46	1.56E-04	1.92E-03
OTU_46 *Incertae Sedis*	HC vs UCD	192.02	–0.67	1.88E-01	3.69E-01
	HC vs TCD	480.32	1.09	1.38E-02	6.17E-02
	UCD vs TCD	512.68	2.34	1.94E-04	2.24E-03
OTU_63 *Blautia*	HC vs UCD	154.0	–0.70	2.24E-01	4.02E-01
	HC vs TCD	341.42	1.69	4.03E-04	3.14E-03
	UCD vs TCD	415.46	2.33	2.2E-04	2.43E-03
OTU_176 *Lachnoclostridium*	HC vs UCD	10.20	–0.71	1.39E-01	3.04E-01
	HC vs TCD	19.53	1.37	8.38E-04	5.89E-03
	UCD vs TCD	21.77	2.03	2.17E-04	2.43E-03
OTU_134 *Ruminiclostridium*	HC vs UCD	30.41	–1.31	9.4E-03	4.59E-02
	HC vs TCD	46.6	0.72	7.65E-02	1.94E-01
	UCD vs TCD	45.40	2.02	2.77E-04	2.83E-03
OTU_136 [*Eubacterium*]	HC vs UCD	117.66	–1.32	5.59E-02	1.57E-01
*coprostanoligenes* group	HC vs TCD	158.56	0.38	4.9E-01	6.54E-01
	UCD vs TCD	218.81	2.38	1.57E-03	1.13E-02
OTU_245 *Christensenellaceae*	HC vs UCD	11.55	–0.64	2.71E-01	4.57E-01
R-7 group	HC vs TCD	23.45	1.52	1.55E-03	9.45E-03
	UCD vs TCD	25.55	2.00	2.23E-03	1.54E-02

NOTE. Log2-fold change is negative when the second group in the pairwise comparison has a lower relative abundance than the first group. n/a, not applicable.

**Supplementary Table 4:** OTUs With Significantly Different Relative Abundance in fecal samples of Paired Data From 13 CD Children at Diagnosis and at 6 and 12 Months After the Initiation of a GFD and From Cross-Sectional Data From UCD Patients (n = 20) and TCD Patients (n = 45)

	Group Comparison	Base Mean	Log2-fold Change	*P*	*P* (Adjusted)
OTU_576 *Akkermansia*	UCD vs GFD 6 mo	212.2	–8.53	1.79E-05	1.27E-03
	UCD vs GFD 12 mo	109.1	–7.00	3.20E-06	1.31E-03
	UCD vs TCD	124.9	1.19	1.27E-01	2.38E-01
OTU_6 *Dialister*	UCD vs GFD 6 mo	1428.3	6.82	1.99E-06	2.83E-04
	UCD vs GFD 12 mo	1283.0	6.88	1.48E-05	1.52E-03
	UCD vs TCD	1540.8	–0.21	7.67E-01	8.48E-01
OTU_5	UCD vs GFD 6 mo	1522.3	–9.80	5.98E-11	1.70E-08
*Phascolarctobacterium*	UCD vs GFD 12 mo	1256.2	–7.66	8.71E-06	1.52E-03
	UCD vs TCD	2385.3	0.83	3.26E-01	4.69E-01
OTU_537 *Dialister*	UCD vs GFD 6 mo	25.8	6.82	2.51E-05	1.42E-03
	UCD vs GFD 12 mo	25.9	7.69	1.15E-05	1.52E-03
	UCD vs TCD	4.68	0.23	3.11E-01	7.29E-01
OTU_8 *Ruminococcus* 2	UCD vs GFD 6 mo	2208.9	4.79	6.18E-03	4.46E-02
	UCD vs GFD 12 mo	3458.3	6.24	1.71E-04	1.40E-02
	UCD vs TCD	2692.5	0.21	7.48E-01	8.33E-01
OTU_43 *Veillonella*	UCD vs GFD 6 mo	343.6	4.80	6.14E-04	1.43E-02
	UCD vs GFD 12 mo	1222.8	5.26	3.85E-04	2.26E-02
	UCD vs TCD	367.3	–0.42	4.66E-01	6.00E-01
OTU_55 [*Eubacterium*]	UCD vs GFD 6 mo	119.2	6.46	1.39E-03	2.28E-02
*coprostanoligenes* group	UCD vs GFD 12 mo	129.2	6.76	3.69E-04	2.26E-02
	UCD vs TCD	86.6	–0.38	5.89E-01	7.09E-01
OTU_1045 *Roseburia*	UCD vs GFD 6 mo	98.6	0.85	5.22E-01	8.06E-01
	UCD vs GFD 12 mo	140.7	5.49	5.53E-04	2.52E-02
	UCD vs TCD	276.2	–0.27	6.85E-01	7.87E-01
OTU_50 *Tyzzerella* 4	UCD vs GFD 6 mo	59.9	–6.20	1.22E-04	3.84E-03
	UCD vs GFD 12 mo	62.6	–5.39	5.08E-04	2.52E-02
	UCD vs TCD	55.6	1.74	5.06E-03	3.02E-02
OTU_191 *Christensenellaceae*	UCD vs GFD 6 mo	16.9	6.09	1.01E-04	3.57E-03
R-7 group	UCD vs GFD 12 mo	17.4	6.00	6.65E-04	2.73E-02
	UCD vs TCD	14.4	–0.74	2.07E-01	3.39E-01
OTU_22 *Ruminococcaceae*	UCD vs GFD 6 mo	748.0	3.92	1.29E-03	2.28E-02
UCG-002	UCD vs GFD 12 mo	772.1	6.01	8.30E-04	2.84E-02
	UCD vs TCD	785.6	–0.22	7.35E-01	8.24E-01
OTU_18 *Phascolarctobacterium*	UCD vs GFD 6 mo	732.0	–0.48	7.88E-01	9.85E-01
	UCD vs GFD 12 mo	109.8	–5.38	7.91E-04	2.84E-02
	UCD vs TCD	9.4	0.96	1.36E-01	2.46E-01
OTU_166 *Cronobacter*	UCD vs GFD 6 mo	25.3	–7.90	5.84E-06	5.53E-04
	UCD vs GFD 12 mo	3.57	–0.02	9.91E-01	9.98E-01
	UCD vs TCD	3.4	0.65	1.54E-01	2.73E-01
OTU_113 *Clostridium* sensu	UCD vs GFD 6 mo	268.6	5.73	7.91E-05	3.21E-03
stricto 1	UCD vs GFD 12 mo	297.8	4.92	2.03E-03	6.43E-02
	UCD vs TCD	148.6	0.67	2.78E-01	4.25E-01
OTU_259	UCD vs GFD 6 mo	26.8	–6.95	7.37E-05	3.21E-03
*Erysipelatoclostridium*	UCD vs GFD 12 mo	10.5	–1.98	2.78E-01	9.98E-01
	UCD vs TCD	8.6	2.61	4.31E-07	1.64E-05
OTU_24 *Lachnoclostridium*	UCD vs GFD 6 mo	1315.9	5.22	2.79E-04	7.91E-03
	UCD vs GFD 12 mo	1471.1	2.51	9.34E-02	6.32E-01
	UCD vs TCD	939.0	–0.70	2.28E-01	2.83E-02
OTU_14 *Subdoligranulum*	UCD vs GFD 6 mo	1006.0	4.16	6.55E-04	1.43E-02
	UCD vs GFD 12 mo	1258.2	2.21	1.30E-01	7.30E-01
	UCD vs TCD	1446.3	1.24	2.59E-02	9.09E-02
OTU_83 *Alistipes*	UCD vs GFD 6 mo	93.9	5.52	6.50E-04	1.43E-02
	UCD vs GFD 12 mo	113.2	5.40	9.00E-03	1.83E-01
	UCD vs TCD	132.2	0.65	3.06E-01	4.49E-01
OTU_66 *Ruminococcaceae*	UCD vs GFD 6 mo	558.9	4.49	7.20E-04	1.46E-02
UCG-005	UCD vs GFD 12 mo	505.6	2.38	1.64E-01	7.98E-01
	UCD vs TCD	304.6	0.77	1.55E-01	2.73E-01
OTU_98 *Ruminococcaceae*	UCD vs GFD 6 mo	57.0	–3.66	8.64E-04	1.63E-02
	UCD vs GFD 12 mo	53.1	–1.81	2.57E-01	9.98E-01
	UCD vs TCD	139.2	2.06	1.39E-04	1.80E-03
OTU_3 *Akkermansia*	UCD vs GFD 6 mo	1033.2	5.40	1.45E-03	2.28E-02
	UCD vs GFD 12 mo	1582.8	2.19	2.35E-01	9.95E-01
	UCD vs TCD	2466.0	1.45	5.12E-02	1.43E-01
OTU_129 *Eggerthella*	UCD vs GFD 6 mo	73.1	–3.76	2.99E-03	3.14E-02
	UCD vs GFD 12 mo	72.6	–1.69	2.12E-01	9.49E-01
	UCD vs TCD	70.16	0.53	2.89E-01	4.36E-01
OTU_893 [*Eubacterium*]	UCD vs GFD 6 mo	82.7	–2.95	2.60E-03	3.14E-02
*hallii* group	UCD vs GFD 12 mo	85.3	–1.93	8.97E-02	6.32E-01
	UCD vs TCD	50.2	0.48	3.50E-01	4.91E-01
OTU_303 *Lachnospiraceae*	UCD vs GFD 6 mo	46.0	–3.69	2.90E-03	3.14E-02
UCG-008	UCD vs GFD 12 mo	62.5	–0.42	7.67E-01	9.98E-01
	UCD vs TCD	114.8	1.47	3.77E-03	2.47E-02
OTU_115 *Parasutterella*	UCD vs GFD 6 mo	56.8	4.42	2.33E-03	3.14E-02
	UCD vs GFD 12 mo	73.4	3.26	4.43E-02	4.54E-01
	UCD vs TCD	36.5	1.62	5.14E-03	3.03E-02
OTU_1005 *Fusicatenibacteri*	UCD vs GFD 6 mo	9.71	–4.70	2.31E-03	3.14E-02
	UCD vs GFD 12 mo	8.7	–2.47	1.28E-01	7.30E-01
	UCD vs TCD	4.25	0.11	8.07E-01	8.76E-01
OTU_133 *Flavonifractor*	UCD vs GFD 6 mo	57.0	–3.64	4.45E-03	4.36E-02
	UCD vs GFD 12 mo	59.1	–2.48	1.07E-01	6.58E-01
	UCD vs TCD	99.5	1.02	5.73E-02	1.55E-01
OTU_1044 [*Eubacterium*]	UCD vs GFD 6 mo	150.3	–3.14	5.18E-03	4.37E-02
*hallii* group	UCD vs GFD 12 mo	149.9	–2.11	1.40E-01	7.58E-01
	UCD vs TCD	152.3	0.04	9.38E-01	9.61E-01
OTU_185 *Dorea*	UCD vs GFD 6 mo	19.4	4.18	5.41E-03	4.37E-02
	UCD vs GFD 12 mo	20.0	3.10	7.03E-02	5.77E-01
	UCD vs TCD	6.5	–0.05	9.29E-01	9.56E-01
OTU_90 *Ruminococcaceae*	UCD vs GFD 6 mo	103.6	4.71	5.05E-03	4.37E-02
UCG-002	UCD vs GFD 12 mo	92.9	3.83	4.53E-02	4.54E-01
	UCD vs TCD	83.8	1.83	1.89E-03	1.34E-02
OTU_122 *Ruminiclostridium* 9	UCD vs GFD 6 mo	25.3	–4.32	5.09E-03	4.37E-02
	UCD vs GFD 12 mo	83.1	–4.10	1.70E-02	2.79E-01
	UCD vs TCD	16.9	0.20	7.33E-01	8.23E-01
OTU_15 *Dorea*	UCD vs GFD 6 mo	275.7	3.58	6.28E-03	4.46E-02
	UCD vs GFD 12 mo	446.8	2.15	1.67E-01	7.98E-01
	UCD vs TCD	538.8	0.82	1.62E-01	2.81E-01
OTU_68 *Ruminococcaceae*	UCD vs GFD 6 mo	152.6	5.45	6.19E-03	4.46E-02
UCG-005	UCD vs GFD 12 mo	77.5	3.89	4.99E-02	4.88E-01
	UCD vs TCD	150.1	1.36	4.10E-02	1.24E-01

NOTE. Log2-fold change is negative when the second group in the pairwise comparison has a lower relative abundance than the first group.

**Supplementary Table 5:** OTUs With Significantly Different Relative Abundance in Fecal Samples of 18 TCD Children and Their Unaffected 19 Siblings

	Base Mean	Log2-fold Change	*P*	*P* (Adjusted)	Higher in
OTU_38 [Eubacterium] *coprostanoligenes* group	75.9	5.27	3.09E-12	1.88E-09	Siblings
OTU_107 *Prevotella* 7	42.2	5.05	7.62E-10	2.32E-07	Siblings
OTU_111 *Blautia*	34.0	4.17	4.98E-09	1.01E-06	Siblings
OTU_62 *Ruminococcaceae* UCG-014	19.1	4.01	3.05E-08	4.65E-06	Siblings
OTU_114 *Coprococcus* 2	12.9	3.85	9.00E-08	1.10E-05	Siblings
OTU_568 *Bifidobacterium bifidum* NCIMB 41171	232.0	4.26	2.10E-07	1.72E-05	Siblings
OTU_135 *Holdemanella*	10.6	3.39	2.26E-07	1.72E-05	Siblings
OTU_143 *Ruminococcus* 1	9.9	3.51	2.18E-07	1.72E-05	Siblings
OTU_295 *Christensenellaceae* R-7 group	6.8	3.43	4.18E-07	2.83E-05	Siblings
OTU_1059 [*Eubacterium*] *coprostanoligenes* group	8.5	3.61	2.93E-06	1.79E-04	Siblings
OTU_228 *Faecalibacterium*	6.5	3.36	3.61E-06	2.00E-04	Siblings
OTU_212 *Ruminiclostridium* 9	7.5	2.73	6.28E-06	3.06E-04	Siblings
OTU_74 [Eubacterium] *ruminantium* group	5.7	3.18	6.53E-06	3.06E-04	Siblings
OTU_78 *Ruminiclostridium* 5	11.2	–3.08	1.11E-05	4.60E-04	TCD
OTU_140 *Collinsella*	6.9	3.13	1.13E-05	4.60E-04	Siblings
OTU_230 *Bacteroides*	6.8	–2.84	1.21E-05	4.62E-04	TCD
OTU_299 [*Ruminococcus*] *gauvreauii* group	9.2	–2.72	1.71E-05	6.07E-04	TCD
OTU_324 *Peptococcaceae*	4.7	2.60	1.79E-05	6.07E-04	Siblings
OTU_88 *Bacteroides*	954.3	–2.89	1.95E-05	6.23E-04	TCD
OTU_665 *Ruminococcaceae* UCG-014	9.1	3.26	2.20E-05	6.68E-04	Siblings
OTU_130 *Christensenellaceae* R-7 group	10.1	2.72	2.67E-05	7.39E-04	Siblings
OTU_576 *Akkermansia*	28.4	–3.03	3.18E-05	8.43E-04	TCD
OTU_80 *Ruminococcaceae* UCG-014	5.7	2.77	3.39E-05	8.60E-04	Siblings
OTU_70 *Ruminococcaceae* UCG-014	5.2	2.75	4.15E-05	1.01E-03	Siblings
OTU_178 *Ruminococcaceae*	8.4	2.42	4.96E-05	1.16E-03	Siblings
OTU_42 *Methanobrevibacter*	17.2	3.21	5.55E-05	1.25E-03	Siblings
OTU_908 *Anaerostipes*	4.3	2.50	7.12E-05	1.46E-03	Siblings
OTU_85 *Bacteroidales* S24-7 group	6.5	–2.68	7.21E-05	1.46E-03	TCD
OTU_272 [*Eubacterium*] *oxidoreducens* group	3.4	2.35	7.14E-05	1.46E-03	Siblings
OTU_180 *Ruminiclostridium* 5	6.2	2.71	8.01E-05	1.57E-03	Siblings
OTU_442 *Christensenellaceae*	3.7	2.44	9.44E-05	1.80E-03	Siblings
OTU_163 *Ruminiclostridium* 5	26.9	–2.34	1.12E-04	2.07E-03	TCD
OTU_33 *Roseburia*	427.7	–2.68	1.17E-04	2.09E-03	TCD
OTU_622 *Lachnospiraceae* UCG-001	52.9	–2.73	1.26E-04	2.20E-03	TCD
OTU_783 *Bacteroides*	4.4	–2.05	2.31E-04	3.71E-03	TCD
OTU_315 *Christensenellaceae* R-7 group	8.9	2.40	2.25E-04	3.71E-03	Siblings
OTU_337 *Coriobacteriaceae*	7.0	2.45	2.31E-04	3.71E-03	Siblings
OTU_67 *Christensenellaceae* R-7 group	53.2	2.71	2.56E-04	4.00E-03	Siblings
OTU_434 *Christensenellaceae*	3.7	2.19	3.06E-04	4.55E-03	Siblings
OTU_155 *Lachnoclostridium*	23.8	–2.56	3.25E-04	4.71E-03	TCD
OTU_179 *Howardella*	3.6	2.28	4.45E-04	6.30E-03	Siblings
OTU_441 *Thalassospira*	3.8	2.13	4.58E-04	6.34E-03	Siblings
OTU_895 *Barnesiella*	21.4	–2.62	5.37E-04	7.26E-03	TCD
OTU_868 *Bacteroides*	112.1	–2.68	6.00E-04	7.77E-03	TCD
OTU_704 *Collinsella*	91.7	–2.16	6.34E-04	7.88E-03	TCD
OTU_133 *Flavonifractor*	82.0	–2.07	9.31E-04	1.09E-02	TCD
OTU_225 *Butyrivibrio*	3.1	2.03	1.16E-03	1.26E-02	Siblings
OTU_149 *Lachnospiraceae* NK4A136 group	25.9	–2.37	1.55E-03	1.58E-02	TCD
OTU_195 *Bacteroides plebeius* DSM 17135	12.2	–2.48	1.84E-03	1.81E-02	TCD
OTU_116 *Ruminococcaceae* UCG-014	9.2	2.07	2.64E-03	2.40E-02	Siblings
OTU_1105 *Prevotella* 9	192.5	3.03	3.33E-03	2.98E-02	Siblings
OTU_137 *Ruminococcaceae* UCG-014	6.6	2.08	3.48E-03	3.05E-02	Siblings
OTU_1049 *Lachnoclostridium*	181.3	–2.06	4.73E-03	3.60E-02	TCD
OTU_63 *Blautia*	388.5	–2.06	4.87E-03	3.66E-02	TCD
OTU_156 *Christensenellaceae* R-7 group	58.7	2.25	4.94E-03	3.67E-02	Siblings
OTU_1054 *Alistipes*	45.1	–2.01	5.82E-03	4.07E-02	TCD

NOTE. Log2-fold change is negative when the second group in the pairwise comparison has a lower relative abundance than the first group.

**Supplementary Table 6:** Fecal Metabolites in CD Children With or Without Recent Ingestion of Gluten, Based on GIP Results

	GIP Negative (n = 59)	GIP Positive (n = 12)	*P*^a^
Ammonia (*×10*^*–4*^*mg/g*)	11.2 (1.8)	7.7 (6.1)	.043
Free sulfide (*μmol/g*)	0.11 (0.03)	0.08 (0.01)	.228
Total sulfide (*μmol/g*)	1.2 (0.25)	0.8 (0.08)	.121
L-lactic acid (*μg/g*)	106.4 (30.6)	86.4 (6.5)	.750
D-lactic acid (*μg/g*)	80.5 (8.4)	87.1 (7.5)	.925
Total lactic acid (*μg/g*)	186.9 (33.9)	173.5 (9.1)	.414
Acetic acid (*μmol/g*)	110.2 (9.5)	125.7 (6.1)	.338
Propionic acid (*μmol/g*)	22.8 (3.1)	23.0 (1.7)	.956
Butyric acid (*μmol/g*)	19.3 (3.1)	21.0 (2.2)	.675
Valeric acid (*μmol/g*)	2.5 (0.51)	2.5 (0.19)	.856
Isobutyric acid (*μmol/g*)	3.1 (0.42)	3.0 (0.20)	.794
Isovaleric acid (*μmol/g*)	3.2 (0.49)	2.95 (0.22)	.640
Total SCFAs (*μmol/g*)	161.0 (13.3)	178.0 (9.9)	.722

NOTE. Values are mean (SEM). Gluten ingestion indicated by a fecal GIP concentration ≥ 0.156 μg/g.

a Mann-Whitney nonparametric test.
